# A novel hypovirulence-associated Hadaka virus 1 (HadV1-LA6) in *Fusarium oxysporum* f. sp. *cubense*

**DOI:** 10.1128/msphere.00428-24

**Published:** 2024-07-16

**Authors:** Yinfu Lin, Guangqun Pan, Yanhua Qi, Bin Wang, Cheng Jin, Wenxia Fang

**Affiliations:** 1 Institute of Biological Science and Technology, Guangxi Academy of Sciences, Nanning, Guangxi, China; 2 College of Life Science and Technology, Guangxi University, Nanning, Guangxi, China; 3 State Key Laboratory of Mycology, Institute of Microbiology, Chinese Academy of Sciences, Beijing, China; University of Michigan, Ann Arbor, Michigan, USA

**Keywords:** *Fusarium oxysporum *f. sp. *cubense*, mycovirus, Hadaka virus 1, hypovirulence

## Abstract

*Fusarium oxysporum* f. sp. *cubense* (*Foc*) poses a significant threat to banana crops as a lethal fungal pathogen. The global spread of *Foc* underscores the formidable challenges associated with traditional management methods in combating this pathogen. This study delves into the hypovirulence-associated mycovirus in *Foc*. From *Foc* strain LA6, we isolated and characterized a novel member of the *Hadakaviridae* family, named Hadaka virus 1 strain LA6 (HadV1-LA6). HadV1-LA6 comprises 10 genomic RNA segments, with RNA1 to RNA7 sharing 80.9%–95.0% amino acid sequence identity with known HadV1-7n, while RNA8 to RNA10 display significantly lower identity. HadV1-LA6 demonstrates horizontal transmission capabilities in an all-or-none fashion between different *Foc* strains via coculturing. Phenotypic comparisons highlight that HadV1-LA6 significantly reduces the growth rates of its host fungus under cell wall stress and oxidative stress conditions. Importantly, HadV1-LA6 attenuates *Foc*’s virulence in detached leaves and banana plants. This study represents the first introduction of a novel hypovirulence-associated Hadaka virus 1 in *Foc*.

**IMPORTANCE:**

Fusarium wilt of banana (FWB) is a severe fungal disease caused by soil-borne *Fusarium oxysporum* f. sp. *cubense* (*Foc*). Among various strategies, biocontrol emerges as a safe, ecologically friendly, and cost-effective approach to managing FWB. In this study, we focus on exploring the potential of a novel hypovirulent member of hadakavirid, HadV1-LA6. Previous reports suggest that HadV1 shows no apparent effect on the host. However, through phenotypic assessments, we demonstrate that HadV1-LA6 significantly impedes the growth rates of its host fungus under stress conditions. More importantly, HadV1-LA6 exhibits a remarkable capacity to attenuate *Foc*’s virulence in detached leaves and banana plants. Furthermore, HadV1-LA6 could be horizontally transmitted between different *Foc* strains, presenting a promising resource for revealing the molecular mechanism of the interaction between Hadaka virus 1 and its host.

## INTRODUCTION

Bananas are globally renowned as the most extensively exported fresh fruit, valued both in terms of volume and economic significance. They play a pivotal role as a staple food in tropical and subtropical regions. Cultivated across more than 120 countries, key production hubs encompass India, Brazil, China, Tanzania, the Democratic Republic of the Congo, various regions in Asia, the Americas, and Africa. Together, these areas contribute to over 70% of the global banana harvest (FAOSTAT, 2023, https://www.fao.org/faostat/en/#data/FO). In 2021, China, ranking as the third-largest banana producer globally, attained a total output of 11,724.2 million tons of fresh bananas on the mainland (https://data.stats.gov.cn/).

Banana Fusarium wilt, caused by *Fusarium oxysporum* f. sp. *cubense* (*Foc*), stands out as the most severe soilborne fungus disease. It infiltrates the xylem tissues of roots and disseminates through the vascular system of pseudostems, ultimately leading to plant fatality (1). *Foc* is categorized into four physiological races or pathotypes*—Foc* race 1 (*Foc*1), *Foc* race 2 (*Foc*2), *Foc* race 3 (*Foc*3), and *Foc* race 4 (*Foc*4) (2). Notably, *Foc*4 is globally prevalent and has been identified in major banana-producing regions, including the alarming ability to infect nearly all banana species, including the resistant Cavendish cultivar clones, which dominate the current export market with a 98% contribution (1, 2). Despite extensive efforts in conventional control methods, managing banana Fusarium wilt remains challenging. *Foc*’s capability to produce chlamydospores and survive as a nonpathogenic parasite on weeds facilitates its prolonged persistence, even in the absence of a living banana host (3). The asymptomatic nature of infected rhizomes contributes to the inadvertent spread of *Foc*, as they are frequently used as seed pieces. Additionally, *Foc* can travel through soil and running water and adhere to farm implements and machinery (4). While current management strategies emphasize preventing *Foc* spread by employing clean planting material and machinery, quarantining infested farms, and other measures, the continued global dissemination underscores the urgent need for effective and environmentally friendly disease control strategies.

Mycoviruses, commonly known as fungal viruses, exhibit the capability to infect and replicate within major filamentous fungal groups, yeasts, and oomycetes (5, 6). Diverging from typical bacterial, plant, and animal viruses, mycoviruses do not instigate the lysis of fungal cells and seldom induce noticeable symptoms in their fungal hosts. Nevertheless, specific mycoviruses can elicit symptomatic infection, such as hypovirulence and debilitation, rendering them promising candidates as biological control agents (5, 7). Notably, Cryphonectria hypovirus 1 (CHV1), a hypovirus infecting *Cryphonectria parasitica*, has demonstrated successful utilization for the biological control of chestnut blight disease in Europe (8). Similarly, Sclerotinia sclerotiorum hypovirulence-associated DNA virus 1 and Rosellinia necatrix megabirnavirus 1 exhibit potential for controlling diseases caused by their respective hosts (9–11). Moreover, some mycoviruses could convert pathogenic fungi into beneficial endophytes and boost plant immunity (12, 13). Given the multitude of instances where mycoviruses effectively diminish the virulence of fungal pathogens, they emerge as valuable tools for developing biological control strategies against fungal diseases in crop plants.

Mycoviruses exhibit a diverse classification with 41 families and over 590 species, as detailed on the International Committee on Taxonomy of Viruses website (https://ictv.global/vmr). The majority of mycoviruses feature a genome composed of double-stranded RNA (dsRNA) or positive-sense single-stranded RNA (+ssRNA), including reverse-transcribing RNA, with negative-sense single-stranded RNA (–ssRNA) or circular single-stranded DNA genomes being seldom observed (14). A recent addition to the +ssRNA mycovirus group is the hadakavirids, now classified in the newly established family *Hadakaviridae* within the phylum *Pisuviricota* (15). Hadakavirids share close relations with members of the *Polymycoviridae* family and exhibit a phylogenetic affinity to +RNA viruses (15). These mycoviruses possess 7- to 11-segmented +ssRNA genomes with generally conserved terminal sequences. Among these segments, RNAs1–3 encode proteins homologous to those encoded by dsRNAs1–3 of *Polymycoviridae* members, namely an RNA-dependent RNA polymerase (RdRp), a hypothetical protein with unknown functions, and a putative methyltransferase (MTR) (16, 17). A distinguishing feature of hadakavirids is their lack of capsids, making their genomic +ssRNAs and replicative form dsRNAs susceptible to exogenously added ribonuclease in host tissue homogenates (16, 17). While hadakavirids isolated from *F. oxysporum* and *Fusarium nygamai* in Pakistan show no apparent effect on the host, *Colletotrichum fructicola* RNA virus 1 (CfRV1), an unassigned member of *Hadakaviridae* discovered in China, induces a mild growth reduction of the host on potato dextrose agar (PDA) medium and a delay in infection on harvested pear fruits (16–18).

Recently, the diversity of mycoviruses within *Foc* was elucidated through metatranscriptomic analysis. This investigation unveiled the presence of five distinct species of mycoviruses in *Foc*, encompassing ourmia-like virus (+ssRNA, *Botourmiaviridae*), mitovirus (+ssRNA, *Mitoviridae*), endornavirus (+ssRNA, *Endornaviridae*), mymonavirus (-ssRNA, *Mymonaviridae*), and partitivirus (dsRNA, *Partitiviridae*). Additionally, two mycoviruses lineages, alphavirus-like virus (+ssRNA) and negative-stranded RNA virus 2 (−ssRNA), were identified but remain unclassified (19). However, the impact of these mycoviruses on *Foc* is yet to be determined.

In this study, we conducted a comprehensive characterization of a newly identified 10-segmented hadakavirid isolated from *Foc* in China. Phylogenetic analysis unveiled its close relation to Hadaka virus 1, with three putative proteins showing limited similarity to other viral proteins. Moreover, the virus induces a notable growth reduction of *Foc* under cell wall and oxidative stress conditions, as well as attenuated pathogenicity toward the plant host. This discovery offers an opportunity for a deeper comprehension of the interaction between +ssRNA hadakavirids and their fungal hosts.

## RESULTS

### Identification of a novel hadakavirid mycovirus from *Foc* strain LA6

**Fig 1:**
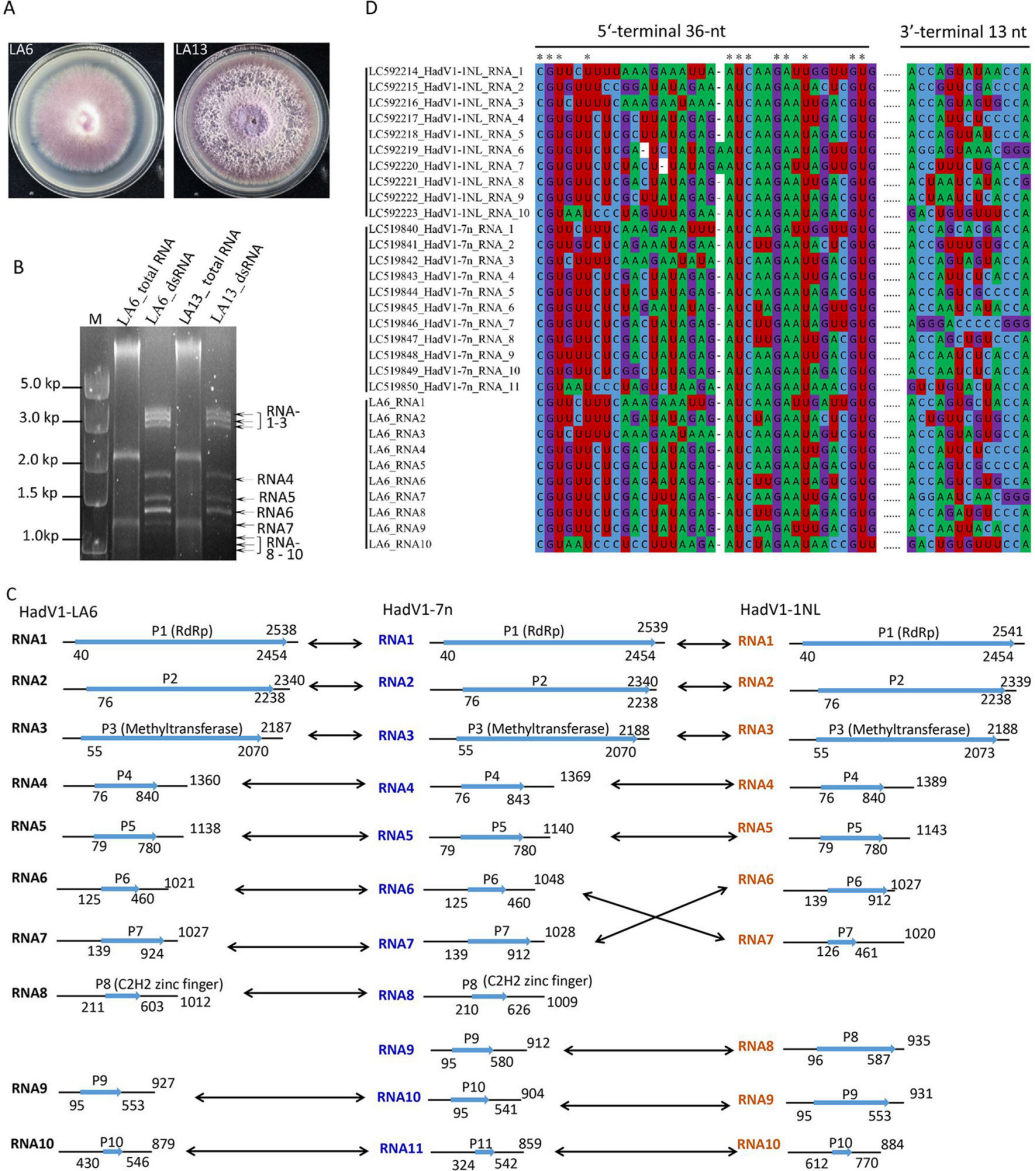
Molecular and phylogenetic characterization of HadV1-LA6. (**A**) Colony morphology of *Foc* strains LA6 and LA13 cultured on PDA medium for 7 days at 28°C. (**B**) Electrophoretic profiles of viral dsRNAs from strains LA6 and LA13 in a 2.0% agarose gel. The dsRNAs were treated with DNase I and S1 nuclease for 1 hour at 37°C. (**C**) A schematic representation of the genomic organization of HadV1-LA6, compared to the genome organization of HadV1-1NL and HadV1-7n. Black arrows indicate pairs of homologous segments conserved among HadV1-LA6, HadV1-1NL, and HadV17n. The light blue arrow indicates the longest open reading frame for each segment. RdRp, methyltransferases, and C_2_H_2_ Zn finger indicate conserved domains such as RNA-dependent RNA polymerase, S-adenosylmethionine-dependent methyltransferases, or a C_2_H_2_ Zn finger motif identified on the genomic fragment. (**D**) Multiple sequence alignment displays the 5′ and 3′-terminal regions of HadV1-LA6 with HadV1-1NL and HadV1-7n genomic segments, visualized using MEGA6 software.

In this study, we conducted a comprehensive virus survey on a collection of 129 *Foc* strains using traditional dsRNA detection methods, and three strains contained dsRNA (Table 1). Gel electrophoresis of the dsRNA revealed a consistent profile with sizes ranging from approximately 1.0 to 3.0 kb in strains LA6 and LA13 (Fig. 1A and B). It is noteworthy that strains LA6 and LA13 were isolated from a healthy banana plant stem and its rhizosphere soil, respectively. Furthermore, unlike *Foc* strain H52, LA6 and LA13 exhibited low pathogenicity in pot experiments involving banana plants (Fig. S1B and C). This observation prompted us to suspect that the dsRNA might be a hypovirulence-inducing virus for *Foc*. To delve deeper into this possibility, we subjected the extracted dsRNAs from strain LA6 to next-generation sequencing (NGS) analysis. After removing low-quality reads and host sequences, the remaining clean reads were *de novo* assembled into 1,335 contigs. These contigs were then screened for sequences similar to known viral sequences using local BLASTn alignment against the GenBank nucleotide database. Our analysis showed that 26 contigs had significant sequence identity to 11 genomic RNA segments of HadV1-7n (*E*-value ≤ 10^–6^) and 10 genomic segments of HadV1-1NL (*E*-value ≤ 10^–5^) (Table S1). These findings strongly imply that the *Foc* LA6 mycovirus is a novel member of the hadakavirid family.

**TABLE 1:** Information of geographical origin and races of *Foc* strains in this study^*a*^

Geographical origin	No. of strains	Isolates from		No. of races			No. of strains that harbor dsRNA
		Plant stem	Rhizosphere soil	Race 1	Race 4	*Foc* ^*a*^	
Fujian	6	6	0	1	5	0	0
Guangdong	20	20	0	4	16	0	0
Hainan	5	5	0	1	4	0	0
Yunnan	2	2	0	1	1	0	0
Guangxi	96	84	12	13	30	53	3
Total	129	117	12	20	56	53	3

^*a*^ Races of *Foc* strains have not been identified.

### Molecular and phylogenetic characterization of HadV1-LA6

The full-length cDNA sequences of the LA6 dsRNA segments were determined by assembling partial-length cDNAs, which were amplified from purified dsRNAs using RT-PCR and the 3′-RLM-RACE protocol (Fig. S2). A total of 10 segments, ranging in size from 880 to 2,538 nucleotides (nt), were obtained and designated as RNA1 to RNA10 in decreasing order of length (Fig. 1C). These sequences were subsequently deposited in GenBank with accession numbers PP501525–PP501534. Notably, the *Foc* LA6 mycovirus lacks either a segment of RNA9 or RNA8, unlike its counterpart hadakavirids HadV1-7n and HadV1-1NL, respectively (Fig. 1C). Similar to its counterpart hadakavirids, all genomic segments exhibit strict conservation of the nucleotide sequence at the 5′ terminal (5′-CGU---) and 3′ terminal (---CCA-3′) ends, with the exception of the presence of “GGG” at the 3′-terminal end of RNA7 (Fig. 1D).

**Fig 2:**
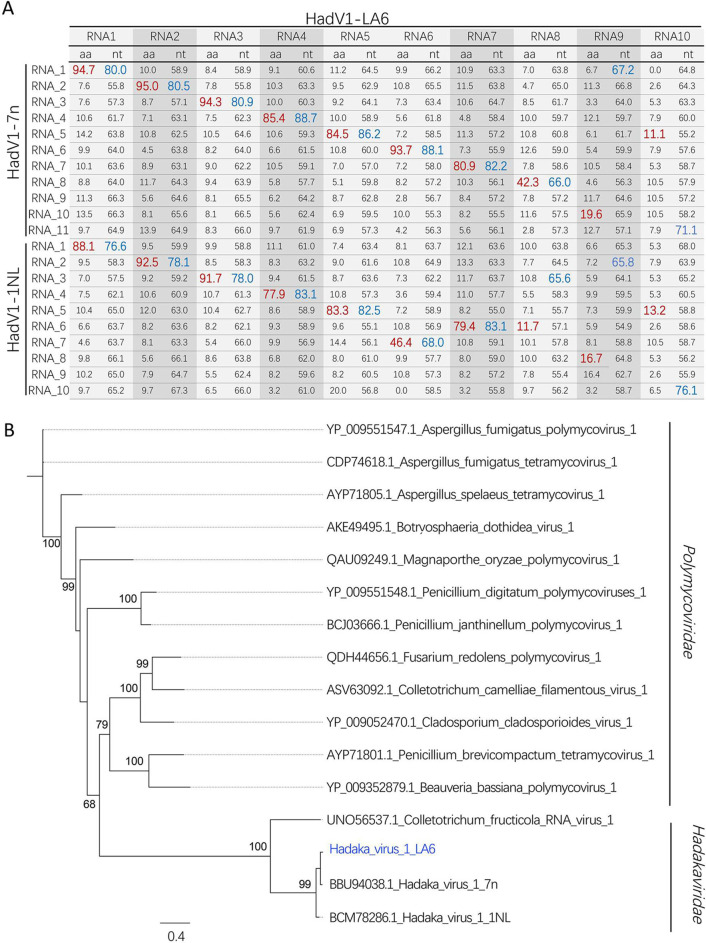
The genetic relationship of HadV1_LA6 with HadV1-1NL and HadV1-7n. (**A**) The highest identities (%) of amino acid (red numbers) and nucleotide (blue numbers) for 10 genome segments of HadV1_LA6 with segments of HadV1-1NL and HadV1-7n. (**B**) Phylogenetic analysis of HadV1_LA6 and related viruses based on RdRp amino acid sequences. A maximum-likelihood phylogenetic tree was generated using MEGA6 with the best-fit model JTT + F + G5 + I. Bootstrap percentages (1,000 replicates) are shown. HadV1_LA6 is marked in blue.

The putative proteins encoded by the *Foc* LA6 mycovirus genome were predicted on the positive strand. The largest open reading frames (ORFs) in RNA1, RNA3, and RNA8 were predicted to encode an RNA-dependent RNA polymerase (P1), a MTR (P3), and a zinc finger protein (P8), respectively. Conversely, the largest ORFs in RNA4, RNA5, RNA6, RNA7, RNA9, and RNA10 encode hypothetical proteins of unknown function (Fig. 1C). A pairwise identity matrix analysis was conducted for nucleotide and amino acid sequences of *Foc* LA6 mycovirus genome with HadV1-7n and HadV1-1N. It was found that RNAs 1–7 exhibited the highest identity at both nucleotide and protein levels with corresponding segments of HadV1-7n followed by HadV1-1NL. However, notable differences were observed for RNAs 8–10 (Fig. 2A). For instance, the sequence identity is low. Although there was high nucleotide identity between *Foc* LA6 mycovirus’s RNA9 with HadV1-7n’s RNA1 and HadV1-1NL’s RNA2, it displayed the highest amino acid identity with HadV1-7n’s RNA10, as well as HadV1-NL’s RNA5, respectively. The smallest fragment, RNA10, shared the highest nucleotide identity with the smallest segment of both HadV1-7n and HadV1-1NL but shared the highest amino acid identity with the RNA5 fragment from both hadakavirids (Fig. 2A). Overall, except for RNA10, which showed higher nucleotide identity with the smallest segment of HadV1-1NL compared to that of HadV1-7n, other fragments from *Foc* mycovirus LA6 demonstrated greater overall similarity in terms of identities when compared to those from HadV1-7n rather than Hadv1-1NL (Fig. 2A).

To examine the phylogenetic relationship between *Foc* LA6 mycovirus and other mycoviruses, the maximum likelihood phylogenetic tree was constructed based on RdRp amino acid sequences of *Foc* LA6 mycovirus and other related viruses from *Polymycoviridae* and *Hadakaviridae*. The results showed that *Foc* LA6 mycovirus grouped within the family *Hadakaviridae*, particularly closer to HadV1-7n (Fig. 2B), which aligns with the findings from the pairwise identity matrix analysis. Based on these results, we conclude that *Foc* LA6 mycovirus is a novel member of the species *Hadakavirus nanga* from the family *Hadakaviridae,* and it is therefore named Hadaka virus 1 strain LA6 (HadV1-LA6).

### HadV1-LA6 is capsidless

To investigate whether HadV1-LA6 shares the capsidless nature observed in hadakavirids such as HadV1-1NL and HadV1-7n, we tested the susceptibility of viral replicative dsRNA forms from LA6 in the mycelial homogenate to RNase A. As a positive control, a mycelial homogenate containing the encapsidated dsRNA virus AfuPmV1 from *Aspergillus fumigatus* isolate Af293 was used. Following treatment with RNase A, the dsRNA bands of HadV1-LA6 became indiscernible in the electrophoretic gel (Fig. S3). In contrast, the quantity and profile of the dsRNA bands of AfuPmV1 seemed unaffected by RNase A treatment (Fig. S3). These findings suggest that the dsRNA replicative form of HadV1-LA6 is also present in a capsidless configuration, similar to other hadakavirids.

### HadV1-LA6 diminishes *Foc* growth under stress conditions

**Fig 3:**
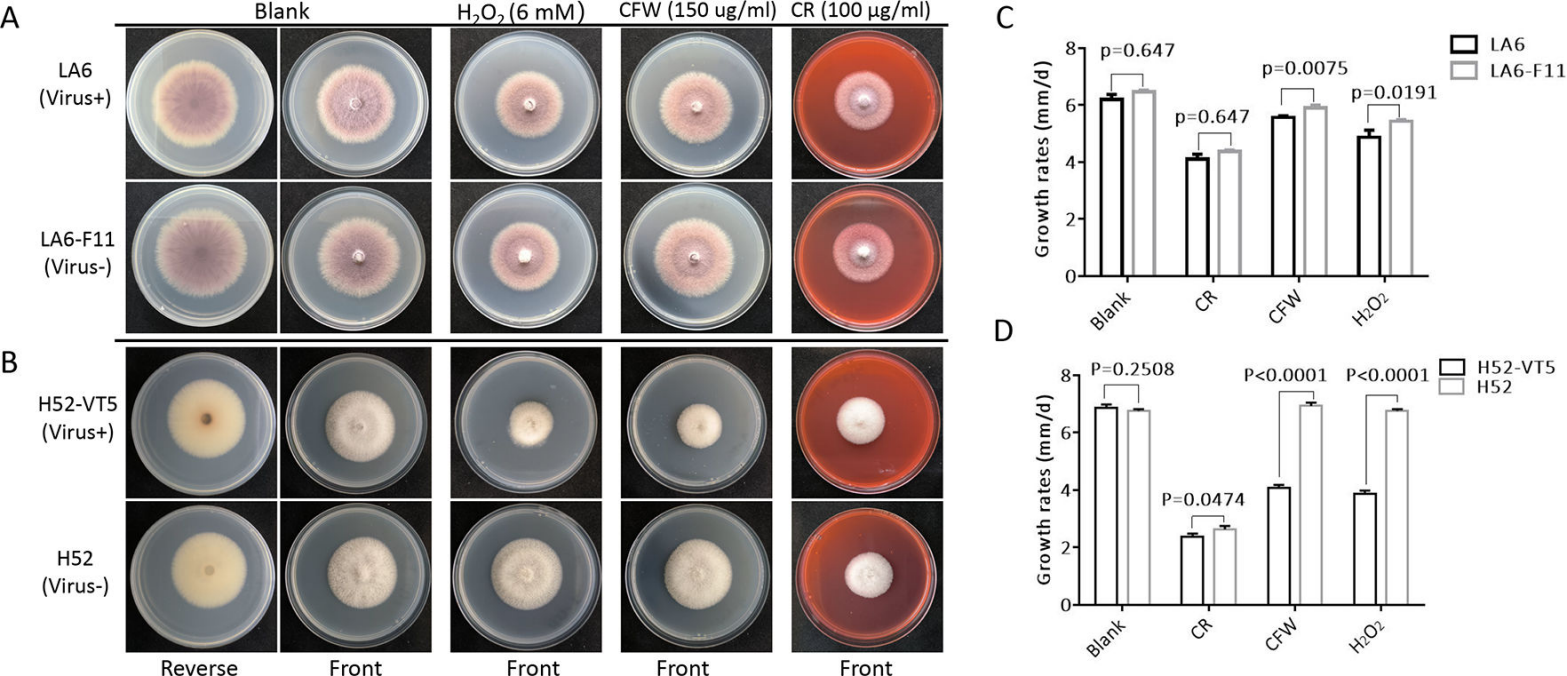
Effects of HadV1_LA6 on fungal morphology and growth. (**A and B**) Representative morphology of the HadV1_LA6-infected *Foc* strain LA6 and virus-cured isolate LA6-F11 (**A**), and horizontal transmission *Foc* strain H52-VT5 and HadV1_LA6-free isolate H52 (**B**) on PDA supplemented with 6 mM H_2_O_2_, 150 µg/mL CFW, and 100 µg/mL CR. (**C and D**) Growth rates of the aforementioned strains; columns indicate the average growth rate of three independent cultures for each subisolate, and error bars represent standard deviation. Unpaired *t*-test was applied for statistical analysis.

To assess the impact of HadV1-LA6 on its fungal host, we generated a virus-cured isogenic subisolate, LA6-F11, derived from *Foc* strain LA6 using a combination of antiviral drug ribavirin treatment and single conidia isolation (Fig. S4A). Additionally, a HadV1-LA6 horizontal transmission subisolate, H52-VT5, was obtained through dual-culturing of H52 (recipient strain) and LA6 (donor strain) (Fig. S5A). No dsRNA bands or RT-PCR products of HadV1-LA6-RNA1 were detected in the virus-cured subisolate LA6-F11 (Fig. S4B and C), while the virus-transmitted subisolate H52-VT5 exhibited an identical dsRNA banding pattern as the donor strain LA6, along with detectable RT-PCR product (Fig. S5B and C). A comparison of HadV1-LA6-infected strains (subisolate) (LA6 and H52-VT5) and virus-free strains (subisolate) (LA6-F11 and H52) revealed no discernible difference in colony morphology and growth rates on PDA plates (Fig. 3A and B). However, when supplemented with H_2_O_2_, calcofluor white (CFW), or Congo red (CR), the growth rates of HadV1-LA6-infected strains were significant slower as compared to the corresponding virus-free strains. The mean difference in growth rate was found to be statistically significant (unpaired *t*-test; *P* value < 0.001) (Fig. 3C and D). Briefly, on the PDA plates supplemented with H_2_O_2_, CFW, and CR, the average growth rates of the horizontal transmission subisolate H52-VT were 3.9, 4.1, and 2.4 mm/day, while those of H52 were 6.8, 6.9, and 2.6 mm/day, respectively; the growth rates for strain LA6 were 4.9, 5.6, and 4.1 mm/day and for cured subisolate LA6-F11 were 5.4, 5.9, and 4.4 mm/day. The difference between H52-VT and H52 was much greater than that between LA6 and LA6-F11 (Fig. 3C and D). Freshly harvested serially diluted conidia (10^5^–10^3^) were point inoculated onto PDA plates supplemented with stress chemicals, and a difference in growth rates between these strains and their subisolates was observed (Fig. S6).

The phenotypic alterations were confirmed by re-introducing HadV1-LA6 into virus-free strains. Two HadV1-LA6 re-infected strains, LA6-F11-T1 and H52-T2, were obtained by co-culturing the HadV1-LA6-free strain LA6-F11 with LA6 and H52 with H52-VT5, respectively (Fig. S7). The growth rates of the HadV1-LA6 re-introduced strains LA6-F11-T1 and H52-T2 were significantly slower compared to the corresponding virus-free strains under stress conditions (Fig. S8).

### HadV1-LA6 mitigates *Foc* pathogenicity

**Fig 4:**
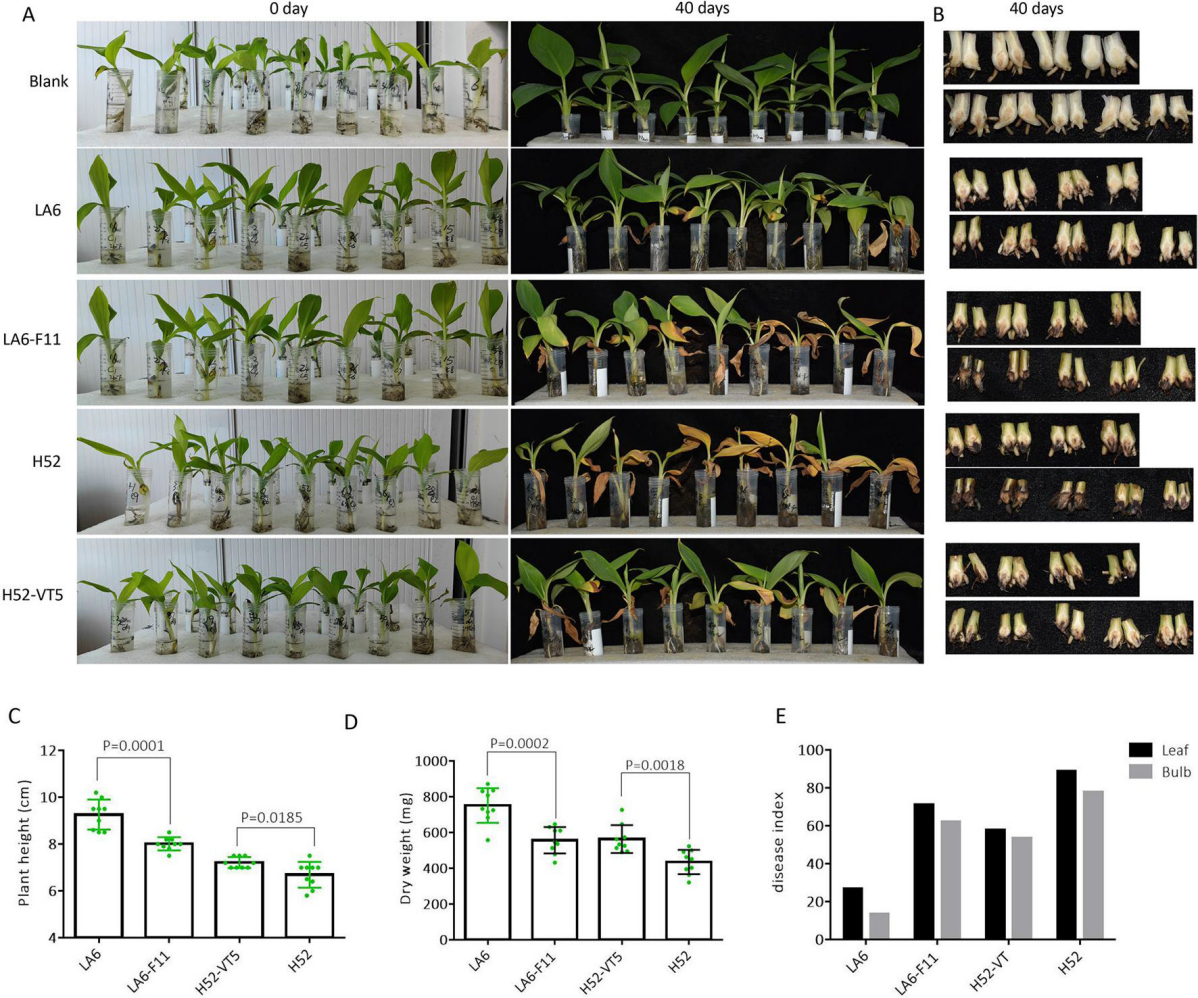
Pathogenicity assessment of the indicated strains in living banana seedlings. (**A**) Leaf wilt symptoms observed in susceptible banana plants inoculated with the HadV1_LA6-infected *Foc* strain LA6, virus-cured isolate LA6-F11, horizontally transmitted *Foc* strain H52-VT5 and HadV1_LA6-free isolate H52, and a blank control (no spores inoculation) for 40 days. (**B**) Bulbs wilt symptoms of the aforementioned banana plants. (**C and D**) Measurement of plant height and dry weight for the mentioned banana plants. Columns represent the average of nine independent experiences, with error bars indicating standard deviation and green dots indicating individual measurements. Statistically significant differences were observed between HadV1_LA6-infected and -free strains (unpaired *t*-test). (**E**) Fusarium wilt index of leaves or bulbs from the aforementioned banana plants was assessed based on disease severity grades.

Pathogenicity assessment of HadV1-LA6-infected strains or subisolates (LA6 and H52-VT5) and virus-free strains or subisolates (H52 and LA6-F11) on detached leaves of banana seedlings indicated that inoculation with virus-free strains or subisolates induced larger lesions compared to virus-infected strains or subisolates (Fig. S9). This strongly suggests that HadV1-LA6 reduces the virulence of *Foc*. Fusarium wilt in banana plants caused by *Foc* is characterized by systemic infection, resulting in typical symptom of leaf wilting, stem and rhizome necrosis. To further determine whether the presence of HadV1-LA6 reduces the virulence of *Foc* in terms of infecting living banana plants, Cavendish banana plantlets were inoculated with *Foc* strains LA6, H52-VT5, LA6-F11, and H52. After 40 days post-inoculation (dpi), plants inoculated with strains LA6 and subisolate H52-VT5 exhibited milder wilt symptoms, including leaf yellowing and bulb browning, compared to those inoculated with LA6-F11 and H52 (Fig. 4A and B). The severity of wilt disease in leaves and bulbs was classified into five grades, where grade 0 indicated no symptoms and grade V indicated severe symptoms (Fig. S10). The Fusarium wilt index was determined based on these grades, showing that virus-infected strains or isolates caused a smaller Fusarium wilt index than virus-free strains or virus-cured subisolates in both leaves and bulbs (Fig. 4E). Although the growth height and dry weight of plants inoculated with HadV1-LA6-infected or -free *Foc* strains or subisolates were all significantly lower than those treated with water, the plants inoculated with strains LA6 or H52-VT5 exhibited significantly greater height and weight compared to plants infected with corresponding virus-free strains (Fig. 4C and D). These results clearly demonstrate that HadV1-LA6 infection attenuates the pathogenicity of *Foc*.

**Fig 5:**
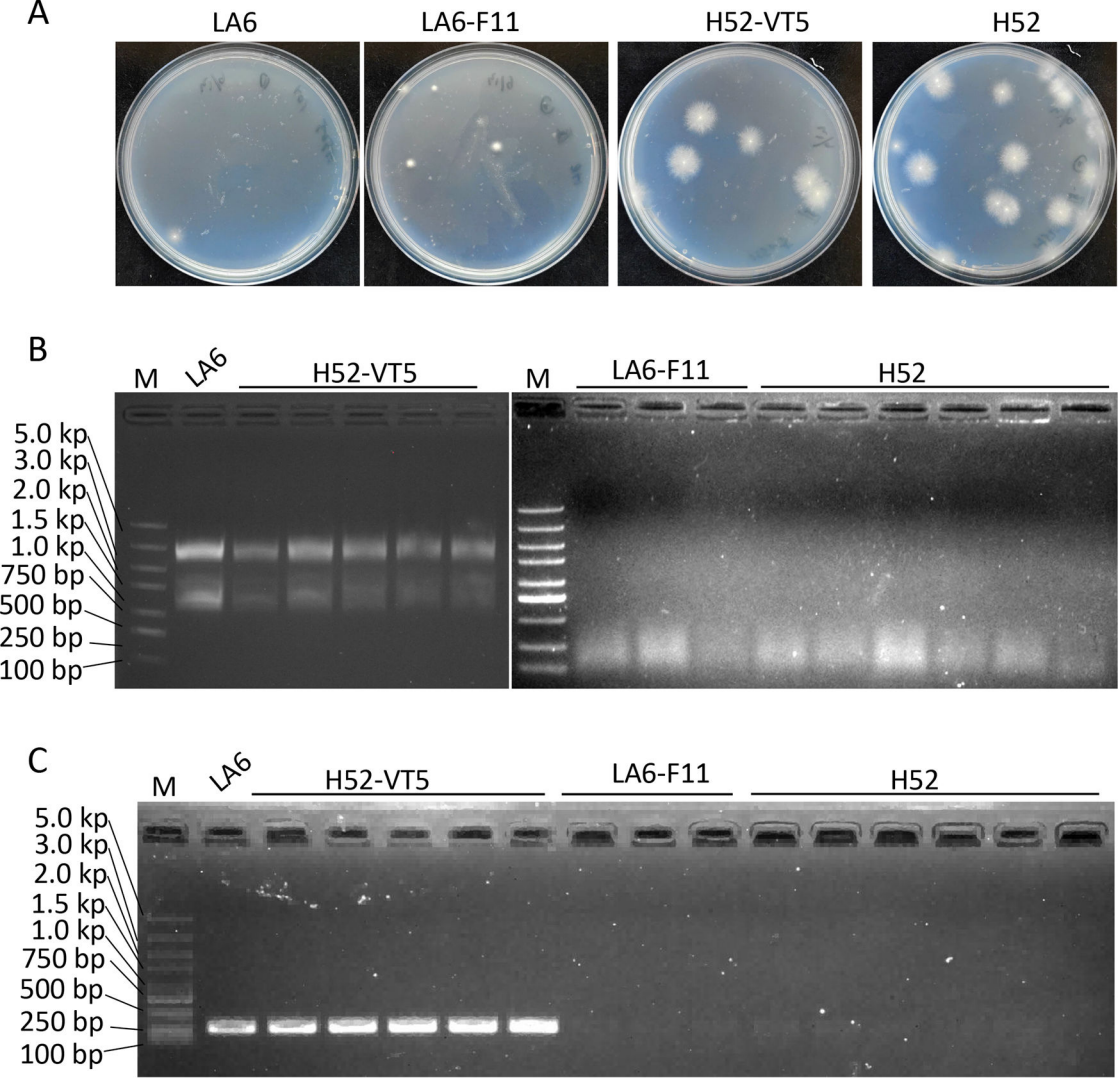
The fungal burden in the rhizomes of banana plantlets infected with *Foc* strains H52, H52-VT5, LA6, and LA6-F11. (**A**) Single colony recovered from the rhizomes of banana plants infected with each indicated strain. (**B and C**) Detection of HadV1_LA6 in *Foc* strains using dsRNA extraction (**B**) and RT-PCR (**C**). M: 5,000 bp DNA ladder.

To evaluate the fungal burden in the rhizomes of H52-, H52-VT5-, LA6-, and LA6-F11-infected banana plantlets, the bulbs were ground, and *Foc* colonies were recovered. A total of 17, 6, 6, and 1 colonies of H52, H52-VT5, LA6-F11, and LA6 were recovered, respectively (Fig. 5A). Compared to the HadV1-LA6 infection group, the *Foc* colony number was significantly higher than the virus-free group, indicating a significant difference in the fungal burden in rhizomes between those inoculated with virus-infected and virus-free strains (Fig. 5A). Furthermore, the exclusive presence of HadV1-LA6 in *Foc* colonies recovered from the bulbs of plants infected with H52-VT5 or LA6, but not in colonies recovered from LA6-F11 or H52-infected plants (Fig. 5B), further confirms that the hypovirulence of *Foc* was attributed to the HadV1-LA6 virus.

## DISCUSSION

Hadakavirids, members of the family *Hadakaviridae*, are recently discovered mycoviruses characterized by a +ssRNA genome. The current taxonomy includes three members: HadV1-7n and HadV1-1NL, both belonging to the species *Hadakavirus nanga* within the genus *Hadakavirus*, and CfRV1, an unassigned member of this group (15). In this study, we characterized a novel member of hadakavirids, designated HadV1-LA6, identified in the banana pathogenic fungus *Foc*. The characterization process involved BLASTn alignment of contigs obtained from NGS (Table S1), construction of phylogenetic trees based on hallmark proteins, particularly P1 (RdRp) (Fig. 2B), and pairwise identity matrix analysis using nucleotide and amino acid sequences of genomes (Fig. 2A). These analyses collectively indicate that HadV1-LA6 is a member of the Hadaka virus 1 and exhibits closer relatedness to isolate HadV1-7n than isolate HadV1-1NL. Consequently, HadV1-LA6 is considered a +ssRNA virus rather than a dsRNA, with the dsRNAs observed on agarose gel (Fig. 1B) identified as its genomic replicative forms.

The taxonomy of capsidless viruses, such as hadakavirids, presents unique challenges compared to encapsidated viruses, where the identification of genomic nucleic acids in virions is straightforward. For example, hypoviruses (family *Hypoviridae*) and endornaviruses (family *Endornaviridae*) were initially classified as dsRNA viruses due to the abundant accumulation of replicative form dsRNA in infected host cells. However, they were later reclassified as +ssRNA viruses based on phylogenetic and evolutionary relationships with members of the expanded picorna-like supergroup viruses with +ssRNA genomes (16, 20, 21). Hadakavirids show a closer phylogenetic affinity to polymycovirids (members of the family *Polymycoviridae*) and the expanded picorna-like supergroup viruses, such as the members of the family *Astroviridae* and *Caliciviridae,* than to dsRNA viruses. Therefore, hadakavirids should be regarded as +ssRNA viruses (15). However, polymycovirids are still classified as dsRNA viruses due to their infectivity through purified dsRNA or PASrp-associated dsRNA (22). Wolf et al. (23) proposed that dsRNA viruses evolved from +ssRNA viruses, whereas −ssRNA viruses originated from dsRNA viruses based on the RdRp phylogenetic tree. Notably, the hallmark GDNQ motif of catalytic core amino acid residues in hadakavirids and polymycovirids differs from the GDD motif found in most dsRNA and +ssRNA viruses. Intriguingly, GDNQ is a hallmark of some negative-sense RNA viruses in the order *Mononegavirales*, such as rhabdoviruses and paramyxoviruses (21). This observation suggests that hadakavirids and polymycovirids may serve as intermediate viruses between typical +ssRNA, dsRNA viruses, and −ssRNA viruses (18, 24). The presence of abundant dsRNA forms of HadV1-LA6 detected through conventional dsRNA detection methods in a hypovirulent *Foc* strain LA6 (Fig. 1A and B), along with the identification of the GDNQ motif in HadV1-LA6 (Fig. S11), further supports this proposal.

Hadakavirids, a group characterized by fluctuating genome segment numbers ranging from 7 to 11 segments (15), include HadV1-LA6 with a 10-segmented genome, likely serving as the exemplar isolate (HadV1-7n) within the *Hadakavirus* genus. Each genome segment encodes a single ORF, commencing with the conserved 5′-CGU and terminating with CCA-3′ (RNA1–RNA6 and RNA8–RNA10) or GGG-3′ (RNA7) (Fig. 1D). Notably, the HadV1-7n’s RNA8 encodes a short C2H2-type zinc finger protein absent in HadV1-1NL (with 10 segments) but present in HadV1-LA6 (Fig. 1D) (16). Conversely, HadV1-LA6 lacks RNA9 compared to HadV1-7n and RNA8 compared to HadV1-1N (Fig. 1D). The segmentation of the viral genome holds significance in genetics and evolutionary biology for RNA viruses (25–27). Fu et al. (18) proposed that varying genomic components in hadakavirids may adapt to different hosts during the evolutionary process. Additionally, among three members of Hadaka virus 1, including HadV1-LA6, the similarity is lower between segments located behind RNA7 compared to those between RNA1 and RNA7 segments. The identity of HadV1-LA6’s RNA8 to RNA9 with corresponding segments of HadV1 is significantly low compared to RNA1 to RNA7 (Fig. 2A). In contrast, CfRVl lacks genomic segments following RNA7. It is speculated that during natural evolution within hadakavirids, these downstream segments of RNA7 may have experienced greater selective pressure, leading them to undergo more changes or adopt strategies such as gene loss as an adaptive response to environmental fluctuations. The smallest fragment in Hadaka virus 1 adds an intriguing aspect; in HadV1-1NL, this segment has a short ORF encoding only 31 amino acids, while in HadV1-7n and HadV1-LA6, it is considerably larger with ORFs encoding 72 and 70 amino acids, respectively. Despite having low identity (<15%) at the level of encoded amino acid sequences for these smallest fragments among all three variants of Hadaka virus 1, their nucleotide identity is surprisingly high (>70%). Another interesting observation is that RNA segments from RNA1 to RNA9 in HadV1-LA6 exhibit higher similarity at both amino acid and nucleotide levels compared to corresponding genomic segments in HadV1-7n; however, this trend reverses for RNA10, which shows higher similarity with HadV1-1NL (Fig. 2A). This suggests potential genome reassortment events between members of Hadaka virus 1, contributing to genomic diversity through segment exchange, similar to what has been described previously for animal and plant RNA viruses (28, 29). Furthermore, the RNA7 segments of HadV1-LA6 and HadV1-7n and the RNA6 segment of HadV1-1NL display a 3′-terminal sequence “---GGG” instead of the strictly conserved “---CCA” observed in the other genomic segments of these three viral strains. This suggests the possibility that this particular genome segment may have originated from another virus with a 3′-terminal sequence of “---GGG.”

Hadakavirids, exemplified by HadV1-7n and HadV1-1NL, deviate from traditional virion structure, hindering their purification through conventional virus purification methods. Their replicative form dsRNAs exhibit susceptibility to exogenously added RNase A in mycelial homogenates, a feature distinct from polymycovirids and encapsidated dsRNA viruses, which tolerate RNase A in the homogenates (15, 16). This study confirmed the capsidless nature of HadV1-LA6 (Fig. S3). Our observations revealed that the 10 genomic segments of HadV1-LA6 can also be horizontally transmitted into another *Foc* strain, H52, via co-culturing in an all-or-none fashion (Fig. S5). However, attempts to transfect H52 protoplasts with HadV1-derived purified dsRNA were unsuccessful (data not shown), leaving the infectious entity of hadakavirids yet to be determined. In contrast, the unassigned member CfRV1 of hadakavirid seems to be associated with the formation of giant vesicles (196–559 nm) containing smaller vesicles (25–40 nm) in host fungal cells (18). Fungal extracellular vesicles (EVs) are involved in the transport of heterogeneous cargos, including proteins, lipids, and nucleic acids, between cells within an organism as well as across kingdoms between microbial pathogens and their hosts (30). We extracted EVs from the virus-infected strain LA6 (Fig. S12A). However, HadV1-LA6 was not detected in the purified EVs using dsRNA extraction or RT-PCR with virus-specific primers (Fig. S12B). This suggests that HadV1-LA6 is not encapsulated within EVs. Nevertheless, we cannot exclude the possibility of HadV1 being encapsulated in other types of vesicles.

Environmental factors play a pivotal role in shaping the outcomes of mycovirus-host interactions, influencing the phenotypic consequences of mycovirus infections. Extensive research has illustrated that various abiotic and biotic factors can either enhance, diminish, or reverse mycovirus-mediated phenotypes (5). Examples include the infection of *Aspergillus thermomutatus* chrysovirus 1, which results in reduced conidiospore production at 20°C but increased production at 37°C compared to uninfected isolates (31). Similarly, the hypovirulence induced by *Magnaporthe oryzae* chrysovirus 1 strain A in rice is dependent on the specific varieties of *M. oryzae*-infected rice (32). Several dsRNA mycoviruses, belonging to families *Polymycoviridae*, *Chrysoviridae*, and *Partitiviridae,* have been reported to render fungal hosts more susceptible to various stresses, including osmotic stress from high salt (33–36); oxidative stress induced by hydrogen peroxide (33, 35–37), high temperature (37), or cell wall-disrupting agents CR treatment (37); bacterial filtrates and volatiles (38); nikkomycin Z (39); and ultraviolet stress (36). In our study, *Foc* infected with HadV1-LA6 exhibited increased sensitivity to specific stresses, such as cell wall-disrupting agents CFW or CR, and oxidative stress induced by H_2_O_2_. Notably, HadV1-LA6 induced asymptomatic infection in *Foc* when tested on a nutrient-rich PDA medium (Fig. 3), consistent with previous findings on HadV1 isolates (15, 16).

Further investigations into the impact of HadV1-LA6 infection on *Foc* under sodium chloride, SDS, and high-temperature exposure did not reveal significant changes in these conditions (data not shown). Stressors like CFW and CR act by binding to nascent chitin chains, inhibiting β-glucan and chitin synthesis, ultimately weakening the cell wall (40). It is plausible that CR or CFW, akin to H_2_O_2_, induces oxidative stress in fungi (37). Oxidative stress can inflict damage and trigger apoptosis in pathogenic fungi (41). Previous studies have shown that mycovirus CHV1 induces oxidative stress in its fungal host *C. parasitica* (42). Similarly, infection with polymycovirids AfuPmV-1 and its variant AfuPmV-1M represses genes involved in *A. fumigatus* defense against oxidative stress. This repression is linked to proteins encoded by dsRNAs 2 and 5, specifically a putative scaffold protein and the protein present in AfuPmV-1M, but not in AfuPmV-1 (33, 37). The response to oxidative stress is also influenced by the putative scaffold protein and the methyltransferase encoded by dsRNA 3 (33, 37). In this context, the infection with HadV1-LA6 may similarly induce oxidative stress or impair responses to oxidative stress, as observed with CHV1 infection or AfuPmV-1/AfuPmV-1M infection. The impact of HadV1-7n and HadV1-NL on the host fungus under PDA medium remains unclear, and whether they also impair the host’s response to stress requires further exploration. Homology between Hadaka virus 1 RNA1, RNA2, and RNA3 and their counterparts in polymycovirids suggests the potential involvement of proteins expressed from dsRNAs 2 and 3 in *Foc*’s response to H_2_O_2_, CFW or CR stresses.

The unassigned member of hadakavirid, CfRV1, has been associated with a mild growth reduction of the host on PDA medium and a delay in infection on harvested pear fruits (18). Similarly, HadV1-LA6 demonstrates attenuated virulence in *Foc*, evident in both detached banana leaves and plants (Fig. 4 and 5). These findings highlight the potential of HadV1-LA6 as a promising source for novel biocontrol agents against banana wilt disease. In plants, exposure to biotic or abiotic stressors can induce the production of reactive oxygen species (ROS), activating various defense mechanisms (43). *Foc* infection is known to trigger high levels of ROS in bananas (44, 45). Moreover, the expression of chitinase and glucanase, both pathogenesis-related (PR) proteins associated with the development of systemic acquired resistance in plants, is consistently upregulated during *Foc* infection (44). These studies collectively suggest that bananas exhibit complex immune responses to *Foc* infection, involving attacks by PR proteins and the activation of ROS signaling pathways. The presence of HadV1-LA6 appears to heighten the sensitivity of *Foc* to these defense reactions in bananas, akin to experimental *in vitro* tests. Additionally, the fungal burden in rhizomes of banana plantlets inoculated with virus-infected *Foc* is significantly lower compared to those inoculated with virus-free *Foc* (Fig. 5A). This indicates that HadV1-LA6 infection diminishes the colonization capacity of *Foc* in banana plants.

Mycoviruses, ubiquitous in fungi, have become more readily discoverable with the advent of next-generation sequencing technology. However, the majority of mycoviruses establish latent infections, and only a small fraction exhibits noticeable symptoms, such as diminished virulence, slow growth rates, and impaired sporulation in their fungal hosts (5, 7). Identifying hypovirulent properties in these mycoviruses is a time-consuming and labor-intensive process. To expedite the identification of hypovirulence-conferring fungal viruses, isolating mycoviruses from endophytic fungi emerges as a rapid and effective method. For instance, chrysovirus-1 was successfully isolated from an endophytic strain of the fungus *Pestalotiopsis theae*, transforming the destructive fungus into a non-pathogenic endophyte in plant hosts (12). In our study, we focused on collecting pseudostems or rhizosphere soil samples from asymptomatic banana plants in *Foc*-infested or intercropped plantations to isolate *Foc* strains. Remarkably, one *Foc* strain isolated from an asymptomatic banana sample in a maize and banana intercropping field (Fig. S1A) housed a new member of the *Hadakaviridae* family (Fig. 2), which significantly attenuated *Foc*’s pathogenicity (Fig. 4). This discovery underscores the potential for endophytes or low-pathogenicity fungal strains to harbor hypervirulent mycoviruses, presenting a promising avenue for the rapid identification of fungal viruses with biocontrol potential.

## MATERIALS AND METHODS

### Fungal strains and culture conditions

A collection of 129 *Foc* isolates underwent mycovirus screening through traditional dsRNA detection (Table 1). Among these *Foc* isolates, 83 were generously provided by Dr. Suiping Huang (*n* = 50) and Prof. Gang Fu (*n* = 33) from Guangxi Academy of Agricultural Sciences. These samples, collected between 2012 and 2016, originated from banana stems in various production areas across China. The remaining isolates, obtained during the present study, were sourced from banana plants or rhizosphere soil samples collected in 2021 from diverse banana cultivation regions in Guangxi province. The confirmation of their identity as *Foc* was achieved through RT-PCR utilizing *Foc*-specific primers (46–48). The primer sets utilized in this investigation are outlined in Table S2. The fungal species identification of strains LA6 and LA13, employed in this study, is shown in Fig. S1D. All strains were stored at −80°C in 25% glycerol and cultured on potato dextrose agar medium at 28°C.

### Nucleic acid and fungal extracellular vesicle extraction

For the extraction of total RNA, total DNA, or dsRNA, *Foc* strains were inoculated onto sterilized cellophane disks on PDA plates and allowed to grow for 5–7 days. Fresh mycelia were harvested and subsequently ground into powder in liquid nitrogen. The extraction of dsRNA was performed by cellulose column chromatography as previously described (49). About 500 ng nucleic acid extracts were further treated with 5 U DNase I (Takara) and 10 U S1 nuclease (Takara) at 37°C for 1 hour. Enzymes were removed through a series of extractions using phenol/chloroform/isoamyl alcohol (25:24:1) followed by a chloroform/isoamyl alcohol (24:1) extraction. The dsRNA was precipitated with ethanol at −20°C overnight and subsequently dissolved in diethyl pyrocarbonate-treated water after centrifugation and drying. Electrophoretic separation of dsRNA was performed with a 1.2% (wt/vol) agarose gel with Tris-acetate-EDTA buffer and visualized by staining with GelRed.

Total DNA was extracted using the CTAB method, while total RNA was extracted using a TransZol up plus RNA kit (Transgen, Beijing, China) according to the manufacturer’s instructions from 100 mg of fresh mycelia. The cDNA was synthesized using HiScript III 1st Strand cDNA Synthesis Kit (+gDNA wiper) (Vazyme, Nanjing, China) with a combination of oligo-dT primers and random primers following the manufacturer’s instructions. PCR was performed using Rapid Taq master mix (Vazyme) mixed with the respective specific primers (Table S2) and 1.0 µL of the fivefold-diluted cDNA products or DNA extracts in a total reaction mixture volume of 20 µL.

The extraction and observation of extracellular vesicles were conducted following previously described methods (30). Briefly, *Foc* mycelia were cultured in PDB media for 72–96 hours at 28°C at 200 rpm. The mycelia were then separated from the culture medium by filtration through clean Miracloth. Spores and cell debris were removed from the culture medium by centrifugation at 4,000 *g* for 15 min followed by 15,000 *g* for 30 min. The resulting supernatant was further subjected to ultra-centrifugation at 100,000 *g* for 1 hour. The EV pellets obtained were resuspended in sterile PBS and subsequently observed under an electron microscope.

### Nucleotide sequencing of viral RNA

The purified dsRNA was sent to Tiangen Biotechnology Inc. (Beijing, China) for next-generation sequencing on an Illumina platform (HiSeqTM2000/MiSeq, 100 bp paired-end reads). A total of 40,474,312 reads were *de novo* assembled into 43,876 contigs using CLC Genomics Workbench version 11 (CLC Bio-QIAGEN, Aarhus, Denmark). Sequence mining was performed using the BLASTN program for nucleic acids or BLASTP for putative proteins against the National Center for Biotechnology Information (NCBI) databases. Virus-sequence-specific primers designed from the contigs obtained from RNA-seq were used for virus detection (Table S2). The nucleotide sequences of the dsRNA’s 5′ and 3′ termini were determined by RNA-ligase-mediated rapid amplification of cDNA ends (3′ RLM-RACE), as described previously (50). Both 3′ termini of dsRNA were ligated with a 3′-RACE adaptor (a 5′-phosphorylated oligodeoxynucleotide: 5′-PO4-CAATACCTTCTGACCATGCAGTGACAGTCAGCATG-3′) at 16°C–18°C. The ligated RNA was used for reverse transcription with the 3′-RACE first primer (5′-CATGCTGACTGTCACTGCAT-3′). The synthesized cDNA was used as a template for PCR with the 3′-RACE second primer (5′-TGCATGGTCAGAAGGTATTG-3′) and each of the segment-specific primers listed in Table S2. The amplified PCR products were purified and cloned into a pUC19 cloning vector using a TA/Blunt-Zero cloning kit (Vazyme) per the manufacturer’s instructions. At least three independent clones of each 3′ RACE product were sequenced using the Sanger method with the M13 universal sequencing primer at AuGct Biotechnology Co. Ltd (Beijing, China).

### Bioinformatic and phylogenetic analysis

NCBI ORF Finder was used to predict the ORFs of the cDNA sequences (https://www.ncbi.nlm.nih.gov/orffinder/). Conserved domains of the putatively encoded proteins were predicted using the Conserved Domain Database (https://www.ncbi. nlm.nih.gov/Structure/cdd/wrpsb.cgi). Multisequence alignment was carried out using the Muscle algorithm (51), as implemented in MEGA6 (Molecular Evolutionary Genetics Analysis version 6) (52). For the construction of maximum-likelihood phylogenetic trees based on deduced amino acid sequences of viral RdRps, MEGA6 was employed, with the substitution model set to general time reversible and 1,000 bootstrap iterations (52). The resultant trees were exported into FigTree v1.4 for viewing and editing (53). Pairwise identity matrix analysis of viral genome sequences (both nucleotide and amino acid) was performed using SDT (Sequeence Demarcation Tool) version 1.2 software (54).

### Curing of virus from strain LA6

To initiate the virus curing process, the hyphae tips were meticulously cut using a sterilized needle and subsequently inoculated onto fresh PDA supplemented with 0.2 mg/mL ribavirin (Cayman Chemical, Ann Arbor, MI, USA) for an additional two or three cycles. Then, the conidia generated on the mycelia were suspended in sterilized water and evenly spread onto PDA medium without ribavirin to generate single conidial subisolates. These subisolates were detected for mycovirus infections using a mycelial direct one-step RT-PCR method as previously described (17). Briefly, mycelial contents were collected on the tip of a toothpick and gently rubbed on the inside bottom of a 0.2 mL PCR tube. Subsequently, the mycelial contents in the tube were mixed with 10 µL of HiScript II One Step RT-PCR Kit (Dye Plus) (Vazyme) containing primers specifically targeting the viral RdRp-encoding segment as listed in Table S1. The RT-PCR was performed following the program: 50°C for 30 min; 94°C for 2 min; 35 cycles of 94°C for 30 s, 60°C for 30 s, and 72°C for 30 s; and a final extension at 72°C for 2 min.

### Horizontal transmission of virus

The transmission of the virus through co-culture involves using the virus-infected strain as the donor and the virus-free strain as the recipient. Mycelial plugs, each with a diameter of 5 mm, were obtained from both the donor and recipient strains and placed 2 cm apart on a 9 cm PDA plate. The plates were then incubated at 28°C for 7 days. Subsequently, mycelial agar plugs were extracted from the edge of each colony of the recipient to obtain derivative isolates. All recipient derivative isolates were analyzed for the infection of virus via dsRNA extraction and RT-PCR.

### Colony growth rate, colony morphology, and conidiation analysis

The growth and colony morphology of both the virus-infected and virus-free strains, as well as subisolates, were evaluated under various stress conditions. PDA plates supplemented with diverse stressors were prepared. Serially diluted conidia (10^5^–10^3^) or mycelial plugs (around 4 mm) were inoculated onto the plates and incubated at 28°C. Colony diameters were measured daily up to 4 dpi using the cross-intersect method, subtracting the diameter of the original disc. The colony morphologies of each strain or subisolate were recorded. For conidiation analysis, conidia from each plate were gently scraped using 0.2% Tween-20 and counted using a hemocytometer.

### Testing the capsidless nature of viral dsRNA

To ascertain the capsidless nature of viral dsRNA, an RNase A treatment was conducted in a crude mycelial homogenate of *Foc* as described previously (17). Briefly, a frozen mycelial culture was ground with a mortar and pestle in liquid nitrogen. The resulting mycelial powder was suspended in 0.05 M sodium phosphate (pH 7.0) and filtered using Miracloth (Merck Millipore). A portion of the mycelial homogenate was then treated with 10 µg/mL RNase A at 37°C for 30 min. The dsRNA was extracted from the mycelial homogenate both before and after RNase A treatment, employing the cellulose column chromatography method, and subsequently analyzed through agarose gel electrophoresis, as described above. *A. fumigatus* strain 293, which harbors the AfuPmV-1 virus, was utilized (55).

### Virulence assay

Two independent methods were employed to assess the virulence of virus-infected *Foc* strains or subisolates on banana plants or detached leaves. First, a pathogenicity assay was conducted on detached leaves from a 30-day-old Cavendish banana cultivar. Briefly, the detached banana leaves were washed thrice with sterile water and air-dried before inoculation. Mycelial plugs with a diameter of 5 mm were placed in the middle of the adaxial surface of wounded detached leaves using a needle (insect pin, 0.45 mm in diameter). Following inoculation. the detached leaves were incubated at 28°C under 99% relative humidity in glass plates with lids. Lesions that developed on the inoculated leaves at 5–7 dpi were measured using ImageJ software. At least three biological replicates for each strain were monitored.

The pathogenicity was also assessed by pot experiments on banana plants. Healthy seedlings of uniform size, with an initial height of 6 cm, were selected, and their roots were cleaned with running water. These selected seedlings were then grown hydroponically in pots containing the necessary nutrient solution (MS), where their roots remained completely immersed in the nutrient solution contained within falcon tubes or grown in boxes filled with sterile soil. At least six plantlets were chosen as replicates, and each *Foc* strain or subisolate was inoculated with 10^7^ spores, while negative control plants received water inoculation only. All inoculated plants were monitored for typical wilt symptom development, and disease symptoms were documented.

After 35 days of growth in sterile soil or 40 days of growth in a nutrient solution, the banana plants were extracted from their respective growing mediums. Subsequently, final measurements of plant height and dry weight, along with the evaluation of disease symptoms, including leaf yellowing and rhizome browning, were conducted. Disease severity was recorded and graded, respectively, from 0 to V for each plantlet. The Fusarium wilt index was assessed according to the following equation:

Disease index = [(Total number of diseased plants of each grade × value of relative grade)/(Total number inspected × 5) ] × 100

The hypovirulence-associated traits of *Foc*, conferred by virus infection, were further validated through the re-isolation of the virus from banana plants in pot experiments. The bulbs from each treatment were separately ground and weighed. Subsequently, the bulbs were surface sterilized with clean water followed by 75% ethanol treatment for 10 s and sodium hypochlorite treatment for 5 min. Next, the tissues were homogenized with quartz sand and PBS buffer to obtain a liquid homogenate. PBS buffer was added to each group based on its weight (3 mL/g). The resulting homogenate was diluted 10-fold and plated onto PDA supplemented with streptomycin using 100 µL from each sample. The plates were incubated at 28°C for 2 days before counting the colonies on each plate. Finally, dsRNA extraction and RT-PCR analysis were performed to detect viruses in individual colonies.

### Statistical analysis

GraphPad Prism 8 software was utilized for curve plotting, and the data were subjected to analysis using an unpaired *t*-test.

## SUPPLEMENTAL MATERIAL

Supplemental figures and tables msphere.00428-24-s0001.docx Tables S1 and S2; Fig. S1 to S12.

ASM does not own the copyrights to Supplemental Material that may be linked to, or accessed through, an article. The authors have granted ASM a non-exclusive, world-wide license to publish the Supplemental Material files. Please contact the corresponding author directly for reuse.
