# Supplementary material for: A novel hypovirulence-associated Hadaka virus 1 (HadV1-LA6) in Fusarium oxysporum f. sp. cubense
Source: mSphere. 2024 Jul 16;9(8):e00428-24. doi: 10.1128/msphere.00428-24 (PMC11351034; doi:10.1128/msphere.00428-24)
Supplement: Supplemental figures and tables — Tables S1 and S2; Fig. S1 to S12. [file msphere.00428-24-s0001.docx]

**Supplementary Information**

**A novel hypovirulence-associated Hadaka virus 1 (HadV1-LA6) in *Fusarium oxysporum* f. sp. *cubense***

Yinfu Lin^a^, Guangqun Pan^a,b^, Yanhua Qi^a^, Bin Wang^a^, Cheng Jin^c^, Wenxia Fang ^a,b^#

^a^Institute of Biological Science and Technology, Guangxi Academy of Sciences, Nanning 530007, Guangxi, China

^b^College of Life Science and Technology, Guangxi University, Nanning 530004, Guangxi, China

^c^State Key Laboratory of Mycology, Institute of Microbiology, Chinese Academy of Sciences, Beijing, China

#Correspondence author’s email: [wfang@gxas.cn](mailto:wfang@gxas.cn)

**Table S1. Results of BLASTN analysis of NGS contigs from LA6 dsRNA.**

| Number | Contigs name | Contig length (nt) | GenBank  accession  number | Accession length (nt) | Accession virus description | Query Cover (%) | E value | Nucleotide identity (%) |
| --- | --- | --- | --- | --- | --- | --- | --- | --- |
| 1^a^ | TRINITY_DN960_c0_g1_i1 | 1572 | NC_078575 | 2539 | Hadaka virus 1 7n genomic RNA, segment 1 | 100 | 0 | 80.36 |
|  |  |  | LC592214 | 2541 | Hadaka virus 1 1NL RNA, segment 1 | 99 | 0 | 78.34 |
| 2^a^ | TRINITY_DN960_c1_g1_i1 | 1017 | NC_078575 | 2539 | Hadaka virus 1 7n genomic RNA, segment 1 | 98 | 0 | 79.68 |
|  |  |  | LC592214 | 2541 | Hadaka virus 1 1NL RNA, segment 1 | 81 | 2.00E-144 | 74.31 |
| 3^a^ | TRINITY_DN881_c0_g1_i1 | 335 | NC_078575 | 2539 | Hadaka virus 1 7n genomic RNA, segment 1 | 99 | 5.00E-88 | 82.63 |
|  |  |  | LC592214 | 2541 | Hadaka virus 1 1NL RNA, segment 1 | 86 | 3.00E-46 | 75.52 |
| 4^a^ | TRINITY_DN460_c0_g1_i1 | 328 | NC_078575 | 2539 | Hadaka virus 1 7n genomic RNA, segment 1 | 100 | 6.00E-49 | 74.09 |
|  |  |  | LC592214 | 2541 | Hadaka virus 1 1NL RNA, segment 1 | 81 | 4.00E-26 | 71.27 |
| 5^a^ | TRINITY_DN913_c0_g1_i1 | 287 | NC_078575 | 2539 | Hadaka virus 1 7n genomic RNA, segment 1 | 99 | 5.00E-62 | 79.72 |
|  |  |  | LC592214 | 2541 | Hadaka virus 1 1NL RNA, segment 1 | 84 | 5.00E-24 | 71.72 |
| 6^a^ | TRINITY_DN1446_c0_g1_i1 | 248 | NC_078575 | 2539 | Hadaka virus 1 7n genomic RNA, segment 1 | 100 | 5.00E-42 | 76.71 |
|  |  |  | LC592214 | 2541 | Hadaka virus 1 1NL RNA, segment 1 | 91 | 3.00E-13 | 70.00 |
| 7^b^ | TRINITY_DN952_c0_g1_i1 | 2308 | NC_078576 | 2340 | Hadaka virus 1 7n genomic RNA, segment 2 | 100 | 0 | 80.66 |
|  |  |  | LC592215 | 2339 | Hadaka virus 1 1NL RNA, segment 2 | 99 | 0 | 78.33 |
| 8^b^ | TRINITY_DN876_c0_g1_i1 | 577 | NC_078576 | 2340 | Hadaka virus 1 7n genomic RNA, segment 2 | 100 | 1.00E-117 | 76.78 |
|  |  |  | LC592215 | 2339 | Hadaka virus 1 1NL RNA, segment 2 | 89 | 3.00E-87 | 74.51 |
| 9^b^ | TRINITY_DN1174_c0_g1_i1 | 562 | NC_078576 | 2340 | Hadaka virus 1 7n genomic RNA, segment 2 | 92 | 1.00E-105 | 77.03 |
|  |  |  | LC592215 | 2339 | Hadaka virus 1 1NL RNA, segment 2 | 99 | 1.00E-80 | 73.67 |
| 10^b^ | TRINITY_DN866_c0_g1_i1 | 490 | LC592215 | 2340 | Hadaka virus 1 7n genomic RNA, segment 2 | 99 | 1.00E-111 | 79.39 |
|  |  |  | NC_078576 | 2339 | Hadaka virus 1 1NL RNA, segment 2 | 99 | 3.00E-99 | 77.39 |

**Table S1 Continued**

| Number | Contigs name | Contig length (nt) | GenBank  accession  number | Accession length (nt) | Accession virus description | Query Cover (%) | E value | Nucleotide identity (%) |
| --- | --- | --- | --- | --- | --- | --- | --- | --- |
| 11^b^ | TRINITY_DN1488_c0_g1_i1 | 485 | NC_078576 | 2340 | Hadaka virus 1 7n genomic RNA, segment 2 | 88 | 2.00E-95 | 79.07 |
|  |  |  | LC592215 | 2339 | Hadaka virus 1 1NL RNA, segment 2 | 96 | 1.00E-73 | 74.15 |
| 12^b^ | TRINITY_DN502_c0_g1_i1 | 469 | LC592215 | 2340 | Hadaka virus 1 7n genomic RNA, segment 2 | 95 | 2.00E-58 | 71.99 |
|  |  |  | NC_078576 | 2339 | Hadaka virus 1 1NL RNA, segment 2 | 95 | 1.00E-41 | 69.13 |
| 13^b^ | TRINITY_DN519_c0_g1_i1 | 287 | NC_078576 | 2340 | Hadaka virus 1 7n genomic RNA, segment 2 | 94 | 6.00E-42 | 75.0 |
| 14^b^ | TRINITY_DN981_c0_g1_i1 | 252 | NC_078576 | 2340 | Hadaka virus 1 7n genomic RNA, segment 2 | 100 | 4.00E-43 | 77.08 |
|  |  |  | LC592215 | 2339 | Hadaka virus 1 1NL RNA, segment 2 | 97 | 1.00E-23 | 71.14 |
| 15^b^ | TRINITY_DN1416_c0_g1_i1 | 215 | NC_078576 | 2340 | Hadaka virus 1 7n genomic RNA, segment 2 | 100 | 7.00E-27 | 74.54 |
|  |  |  | LC592215 | 2339 | Hadaka virus 1 1NL RNA, segment 2 | 72 | 8.00E-20 | 75.95 |
| 16^b^ | TRINITY_DN210_c0_g1_i1 | 207 | LC592215 | 2339 | Hadaka virus 1 1NL RNA, segment 2 | 86 | 1.00E-11 | 71.11 |
| 17^c^ | TRINITY_DN944_c0_g1_i1 | 2234 | NC_078573 | 2188 | Hadaka virus 1 7n genomic RNA, segment 3 | 95 | 0 | 80.74 |
|  |  |  | LC592216 | 2188 | Hadaka virus 1 1NL RNA, segment 3 | 96 | 0 | 80.74 |
| 18^c^ | TRINITY_DN623_c0_g1_i1 | 243 | NC_078573 | 2188 | Hadaka virus 1 7n genomic RNA, segment 3 | 99 | 3.00E-45 | 77.66 |
|  |  |  | LC592216 | 2188 | Hadaka virus 1 1NL RNA, segment 3 | 98 | 3.00E-32 | 74.06 |
| 19^d^ | TRINITY_DN962_c1_g1_i1 | 1341 | NC_078574 | 1367 | Hadaka virus 1 7n genomic RNA, segment 4 | 100 | 0 | 87.20 |
|  |  |  | LC592217 | 1389 | Hadaka virus 1 1NL RNA, segment 4 | 99 | 0 | 83.32 |
| 20^e^ | TRINITY_DN954_c0_g1_i1 | 1137 | NC_078577 | 1140 | Hadaka virus 1 7n genomic RNA, segment 5 | 96 | 0 | 84.76 |
|  |  |  | LC592218 | 1143 | Hadaka virus 1 1NL RNA, segment 5 | 87 | 0 | 80.98 |
| 21^f^ | TRINITY_DN953_c0_g1_i1 | 1025 | LC592219 | 1027 | Hadaka virus 1 1NL RNA, segment 6 | 98 | 0 | 83.3 |
|  |  |  | NC_078578 | 1028 | Hadaka virus 1 7n genomic RNA, segment 7 | 97 | 0 | 82.21 |

**Table S1 Continued**

| Number | Contigs name | Contig length (nt) | GenBank  accession  number | Accession length (nt) | Accession virus description | Query Cover (%) | E value | Nucleotide identity (%) |
| --- | --- | --- | --- | --- | --- | --- | --- | --- |
| 22^g^ | TRINITY_DN921_c0_g1_i1 | 991 | NC_078572 | 1048 | Hadaka virus 1 7n genomic RNA, segment 6 | 82 | 0 | 91.47 |
|  |  |  | LC592220 | 1020 | Hadaka virus 1 1NL RNA, segment 7 | 60 | 8.00E-41 | 68.49 |
| 23^h^ | TRINITY_DN928_c0_g1_i1 | 970 | NC_078570 | 1009 | Hadaka virus 1 7n genomic RNA, segment 8 | 45 | 4.00E-25 | 69.97 |
| 24^i^ | TRINITY_DN955_c1_g2_i1 | 907 | LC592221 | 935 | Hadaka virus 1 1NL RNA, segment 8 | 17 | 4.00E-19 | 85.71 |
|  |  |  | LC519848 | 912 | Hadaka virus 1 7n genomic RNA, segment 9 | 27 | 5.00E-18 | 84.54 |
|  |  |  | LC592222 | 931 | Hadaka virus 1 1NL RNA, segment 9 | 10 | 1.00E-11 | 79.38 |
|  |  |  | NC_078569 | 904 | Hadaka virus 1 7n genomic RNA, segment 10 | 27 | 6.00E-10 | 78.35 |
| 25^i^ | TRINITY_DN716_c0_g1_i1 | 209 | NC_078569 | 904 | Hadaka virus 1 7n genomic RNA, segment 10 | 40 | 3.00E-06 | 77.38 |
|  |  |  | LC592221 | 935 | Hadaka virus 1 1NL RNA, segment 8 | 30 | 3.00E-05 | 81.25 |
| 26^j^ | TRINITY_DN1308_c0_g1_i1 | 899 | LC592223 | 884 | Hadaka virus 1 1NL RNA, segment 10 | 66 | 1.00E-145 | 80.6 |
|  |  |  | NC_078568 | 859 | Hadaka virus 1 7n genomic RNA, segment 11 | 50 | 1.00E-57 | 73.25 |

^a^ to ^j^: Contigs were successfully mapped to 1 to 10 genome segments of the Hadaka virus 1 strain LA6 respectively, which were obtained through 3’-RLM-RACE and RT-PCR using the primer set provided in table S1.

**Table S2. Primers used in this study.**

| **Primer name** | **Detect object** | **Primer sequence (5'to 3')** | **Product size (bp)** | | **Ref.** |
| --- | --- | --- | --- | --- | --- |
| LA6RNA1-1F | HadV1-LA6 RNA1 | TGCAAGATGTCTGGCAAACCAAG | | 304 | This study |
| LA6RNA1-1R | HadV1-LA6 RNA1 | CGTCTGGTACAGACACTTTCATCTCA | | 304 | This study |
| LA6RNA1-2F | HadV1-LA6 RNA1 | GATAGGCGCACGTGCTTATCTTTACTCA | | 242 | This study |
| LA6RNA1-2R | HadV1-LA6 RNA1 | GAGATATCATCCAGGTCAAGGAGGACA | | 242 | This study |
| LA6RNA2-1F | HadV1-LA6 RNA2 | CCAAGTACGGATGGACTTGTCTTCA | | 467 | This study |
| LA6RNA2-1R | HadV1-LA6 RNA2 | CGTACGGTGTATTAGCTCTCA | | 467 | This study |
| LA6RNA2-2F | HadV1-LA6 RNA2 | CGACGATATAATGGATGCAAATGA | | 367 | This study |
| LA6RNA2-2R | HadV1-LA6 RNA2 | TGTGTTAGTAGGTACTAAGCTTGC | | 367 | This study |
| LA6RNA3-1F | HadV1-LA6 RNA3 | TCTCGTTCATCTGTATCGTCA | | 471 | This study |
| LA6RNA3-1R | HadV1-LA6 RNA3 | CCTTCTATTCGTCACCAAACCA | | 471 | This study |
| LA6RNA3-2F | HadV1-LA6 RNA3 | ATCTAGAAGAGGCTGTGTCA | | 368 | This study |
| LA6RNA3-2R | HadV1-LA6 RNA3 | CCATTGTGATAGTAGGTCGA | | 368 | This study |
| LA6RNA4-1F | HadV1-LA6 RNA4 | GTTCAACAGGAGACAAAATGCA | | 328 | This study |
| LA6RNA4-1R | HadV1-LA6 RNA4 | CATCACGTTCGCAATAAGTC | | 328 | This study |
| LA6RNA4-2F | HadV1-LA6 RNA4 | CCTTGGCCTGTACTGGTGT | | 371 | This study |
| LA6RNA4-2R | HadV1-LA6 RNA4 | CGACCCATTCTCTCGTCGGAA | | 371 | This study |
| LA6RNA5-F | HadV1-LA6 RNA5 | CTAACCAGGCCGAGCGTA | | 920 | This study |
| LA6RNA5-R | HadV1-LA6 RNA5 | GGCCCAGTCACATACCGAATA | | 920 | This study |
| LA6RNA6-F | HadV1-LA6 RNA6 | GAGATCTTGAATAGTCGTGCAAGT | | 972 | This study |
| LA6RNA6-R | HadV1-LA6 RNA6 | GCTCAAGTCGAGTGGTACCT | | 972 | This study |
| LA6RNA7-F | HadV1-LA6 RNA7 | CGTGTGCTCTTCCGATCTTTTAGAGA | | 1005 | This study |
| LA6RNA7-R | HadV1-LA6 RNA7 | ACGGGTTCTTATTGGCCTTTCA | | 1005 | This study |
| LA6RNA8-F | HadV1-LA6 RNA8 | GCGTATCCTTTAGAAACCGTCTTCA | | 944 | This study |
| LA6RNA8-R | HadV1-LA6 RNA8 | GAAGCTCGTGGAATCTTCTACAGGA | | 944 | This study |
| LA6RNA9-1F | HadV1-LA6 RNA9 | CTCGACTATAGAGATCAAGATTTGACGT | | 901 | This study |
| LA6RNA9-1R | HadV1-LA6 RNA9 | AGCCTAGACAAACGTGAAACTAAG | | 901 | This study |
| LA6RNA9-2F | HadV1-LA6 RNA9 | CTAAGTGGTTAGTCTACCTTTCATTTGT | | 206 | This study |
| LA6RNA9-2R | HadV1-LA6 RNA9 | GTTGCTGCGAGTTGGTTTACCA | | 206 | This study |
| LA6RNA10-F | HadV1-LA6 | GGTGACTGGAGTTCAGACGT | | 850 | This study |
| LA6RNA10-R | HadV1-LA6 | AGGCAGTGTGCATGAACGA | | 850 | This study |
| W106F | Foc | GCAGTCGTACGTCATCGACC | | 729 | (1) |
| W106R | Foc | CCATGGCAGATGGCGAGTCA | | 729 | (1) |
| *Foc*TR4-F | *Foc* R4 | CACGTTTAAGGTGCCATGAGAG | | 463 | (2) |
| *Foc*TR4-R | *Foc* R4 | CGCACGCCAGGACTGCCTCGTGA | | 463 | (2) |
| SIX6b-210-F | *Foc* R1 | ACGCTTCCCAATACCGTCTGT | | 210 | (3) |
| SIX6b-210-R | *Foc* R1 | AAGTTGGTGAGTATCAATGC | | 210 | (3) |

Foc: *Fusarium oxysporum* f. sp. *cubense*.

*Foc* R1 and *Foc* R4: *Fusarium oxysporum* f. sp. *cubense* physiological race 1 and race 4.

**Supplementary figures and legends**
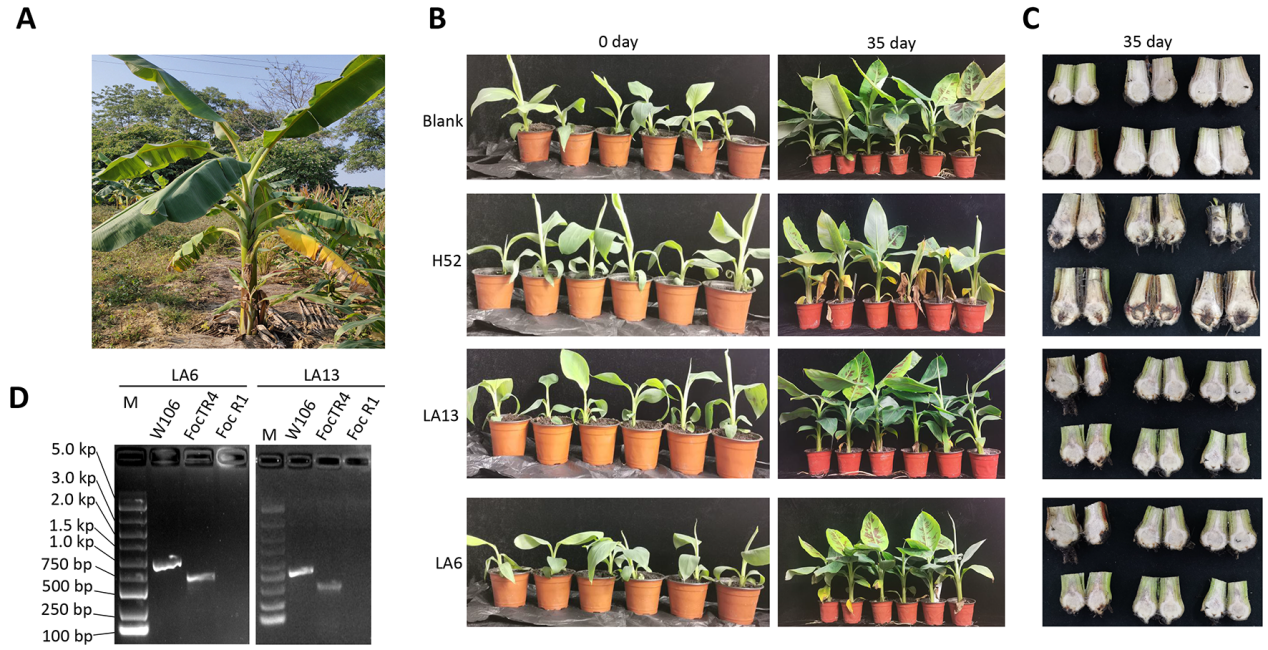


**Fig. S1 Pathogenicity detection of *Foc* strains LA6 and LA13 in living banana seedlings.**

**(A)** a almost free of fusarium wilt banana plant in which strain LA6 was isolated from bulb and strain LA13 isolated from rhizosphere soil.

**(B)** Leaf wilt symptoms from the susceptible banana plants after inoculated 28 days with the strains LA6 and LA13, and a strong virulent strain H52, Blank (no spores). **(C)** Bulbs wilt symptoms of the aforementioned banana plants.

**(D)** Strains of LA6 and LA13 identified by PCR using *Foc* specific primers.


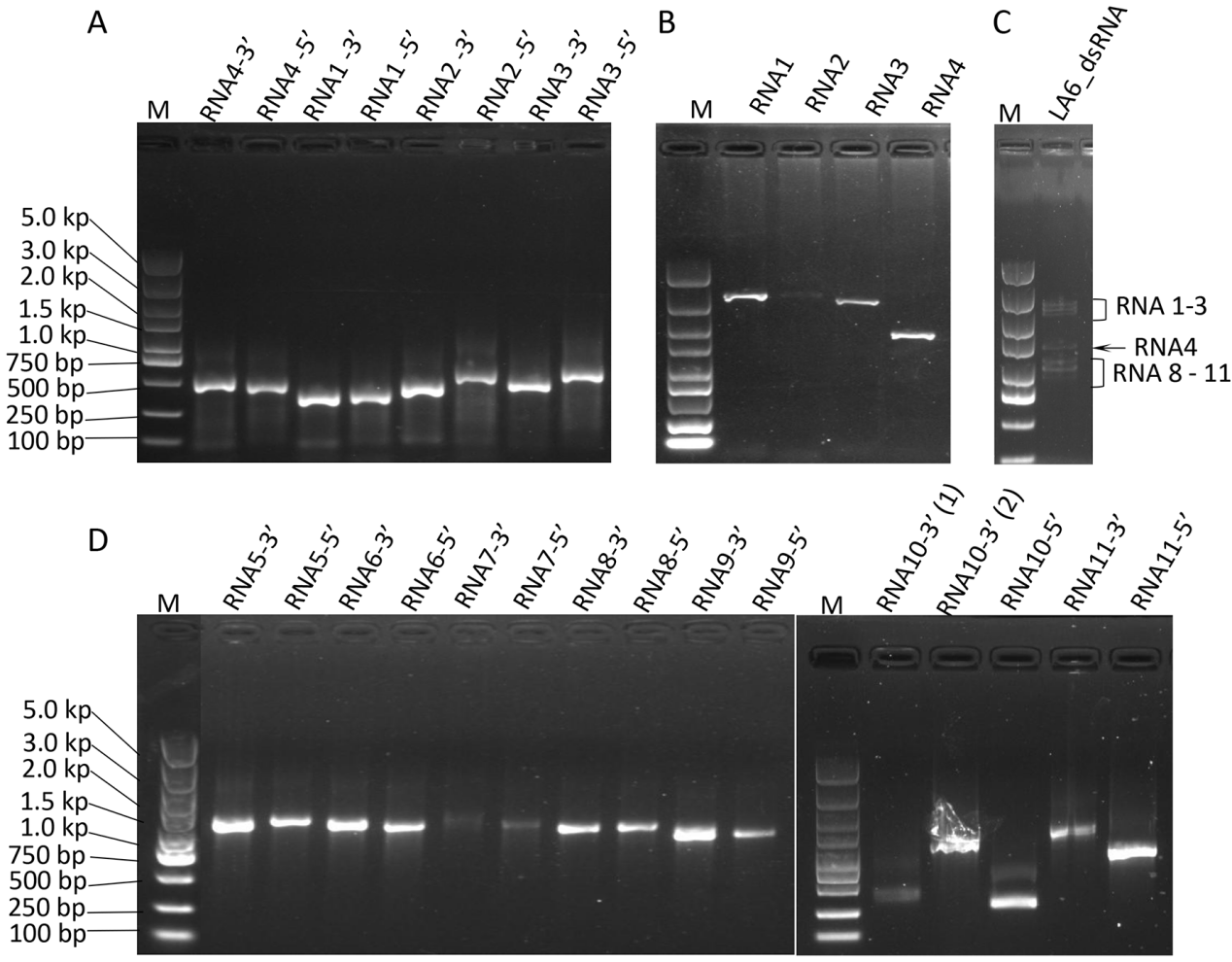


**Fig. S2 The complete genome nucleotide sequence of 10 segments of HadV1_LA6 obtained by RNA-ligase-mediated rapid amplification of cDNA ends (3’RLM-RACE) and RT-PCR.**

**(A and D)** Nucleotide sequences of the 5’ and 3’ termini of the dsRNA segments RNA1 to RNA4, and complete sequence of segments RNA1 to RNA4 were amplified using 3’RLM-RACE method, respectively.

**(B)** The approximately full length of segments RNA1 to RNA4 were amplified using RT-PCR with virus specific primers (Table S1).

**(C)** The dsRNA template for HadV1_LA6 genomic sequence clone.


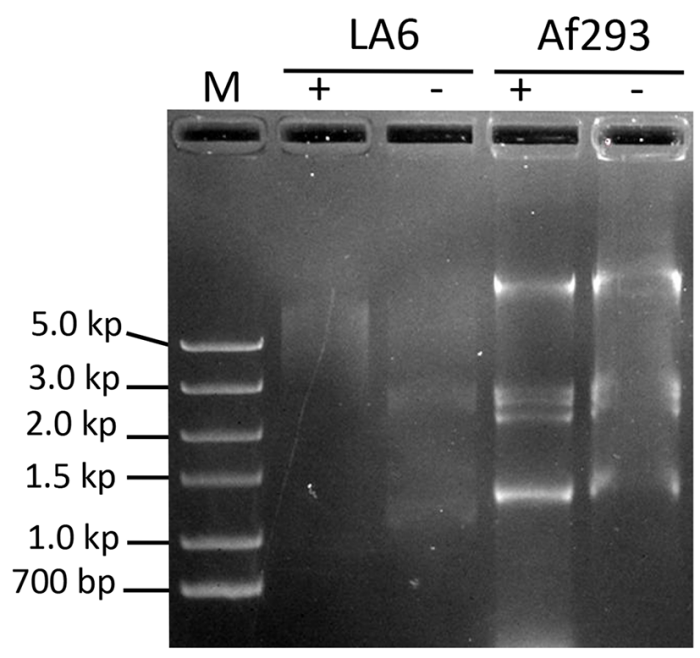


**Fig. S3 RNase A susceptibility of viral dsRNA in mycelial homogenates.** Agarose gel electrophoretic profiles depict viral dsRNA extracted before (-) and after (+) RNaseA treatment. The tested viruses included HadV1_LA6 from *Foc* isolate LA6, and *Aspergillus fumigatus* polymycovirus 1 (AfuPmV1) from *A. fumigatus* strain Af293. AfuPmV1 is a polymycovirid with non-conventionally encapsidated dsRNA genomes.


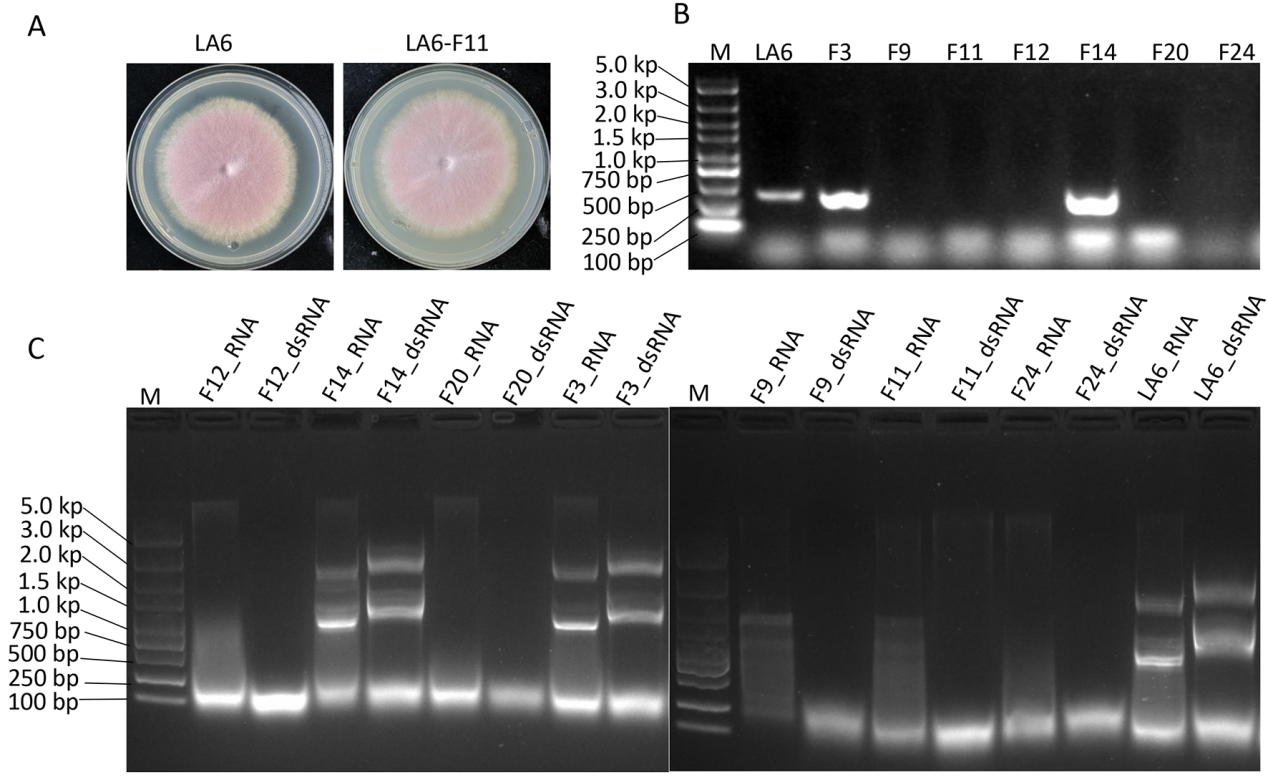


**Fig. S4 Virus curing of *Foc* strain LA6 on PDA plate supplemented with 0.2 mg/ml** **ribavirin.**

**(A)** Colony morphology of LA6 and a virus-cured isolate LA6-F11 on PDA medium for 7 days at 28℃.

**(B and C)** HadV1_LA6 detected of single conidial subisolates from ribavirin treated LA6 using RT-PCR (A) and dsRNA extracting (C).


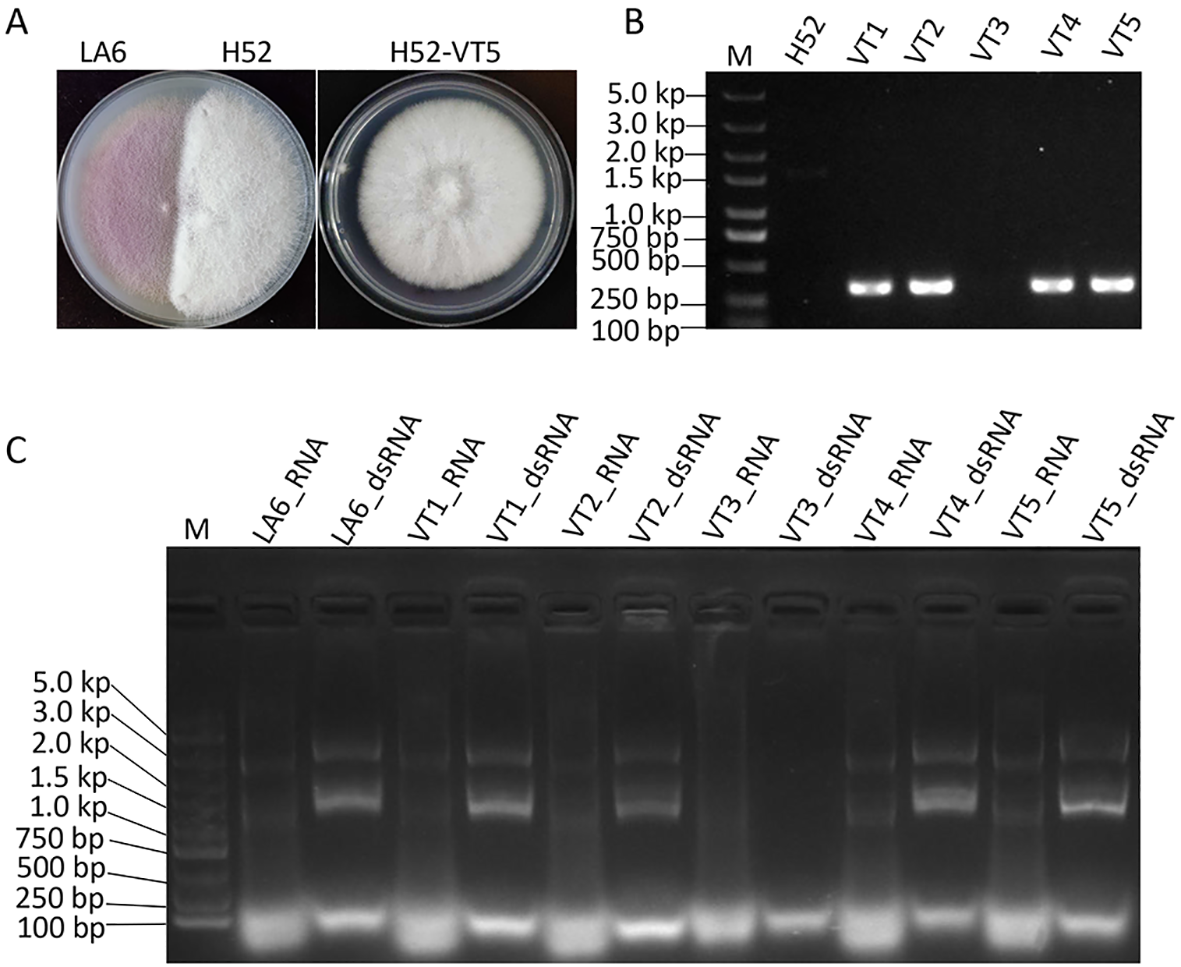


**Fig. S5 HadV1_LA6 horizontal transmission from *Foc* strain LA6 (donor) into *Foc* strain H52 (recipient).**

**(A)** Colony morphology of confrontation culture (LA6 vs H52) and a virus-transmit subisolate H52-VT5 on PDA medium for 6 days at 28℃.

**(B)** HadV1_LA6 detected of subisolates from confrontation culture H52 using RT-PCR.

**(C)** HadV1_LA6 detected of subisolates from confrontation culture H52 using dsRNA extracting.


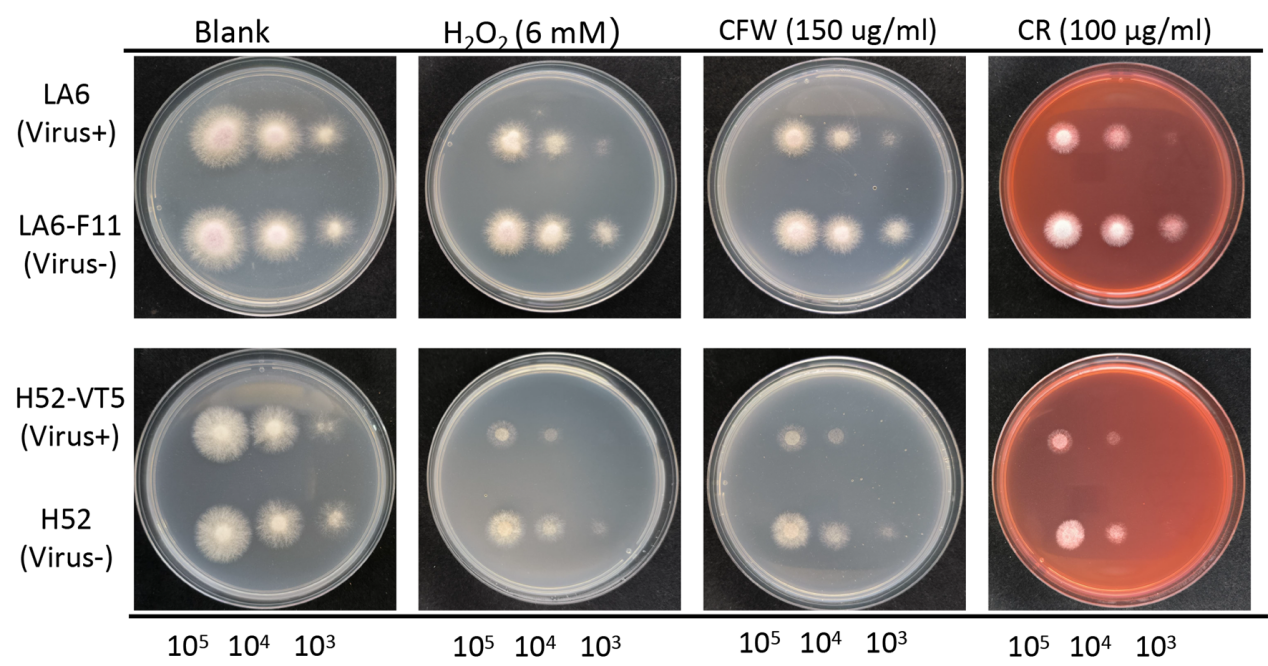


**Fig. S6** **Effects of HadV1_LA6 on *Foc* conidia growth.** Freshly harvested serially diluted conidia (10^5^–10^3^) were point inoculated onto PDA plates supplemented with 6 mM H_2_O_2_, 150 µg/ml CFW and 100 µg/ml CR.


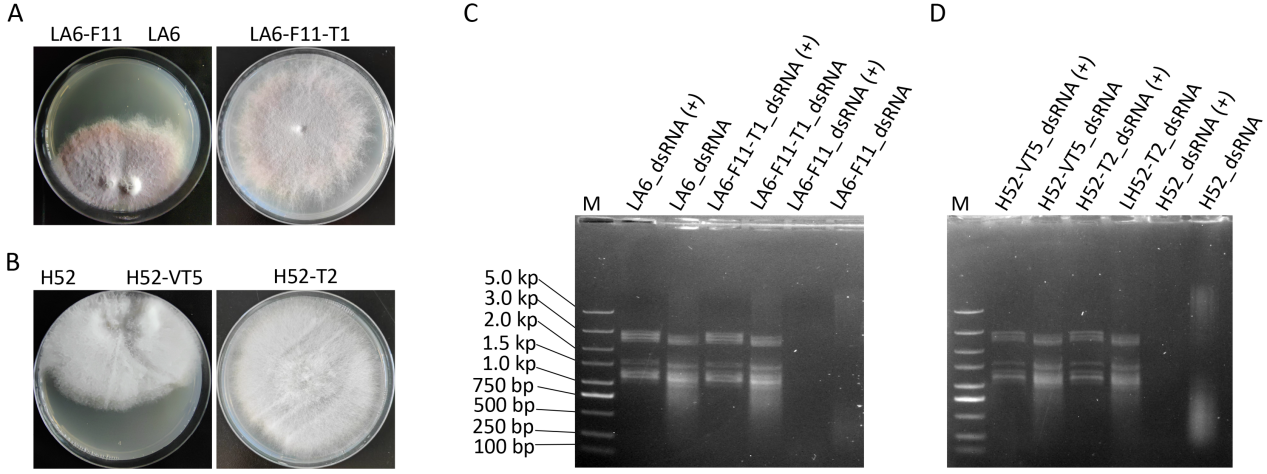


**Fig. S7 Re-introducing of HadV1_LA6 from *Foc* strain LA6 and H52-VT5 into strains LA6-F11 and H52 via co-culture.**

**(A)** Colony morphology of confrontation culture (LA6 vs LA6-F11) and a virus-transmit subisolate LA-F11-T1 on PDA for 7 days at 28℃.

**(B)** Colony morphology of confrontation culture (H52-VT5 vs H52) and a virus-transmit subisolate H52-T2 on PDA for 6 days at 28℃.

**(C)** Detection of HadV1_LA6 in re-introduced strains LA6-F11-T1 using dsRNA extraction. LA6 and LA-F11 are positive and negative control, repectively.

**(D)** Detection of HadV1_LA6 in re-introduced strain H52-T2 using dsRNA extraction. H52-VT2 and H52 are positive and negative control, repectively.

The + indicates that the dsRNA was treated with DNase I and S1 nuclease.


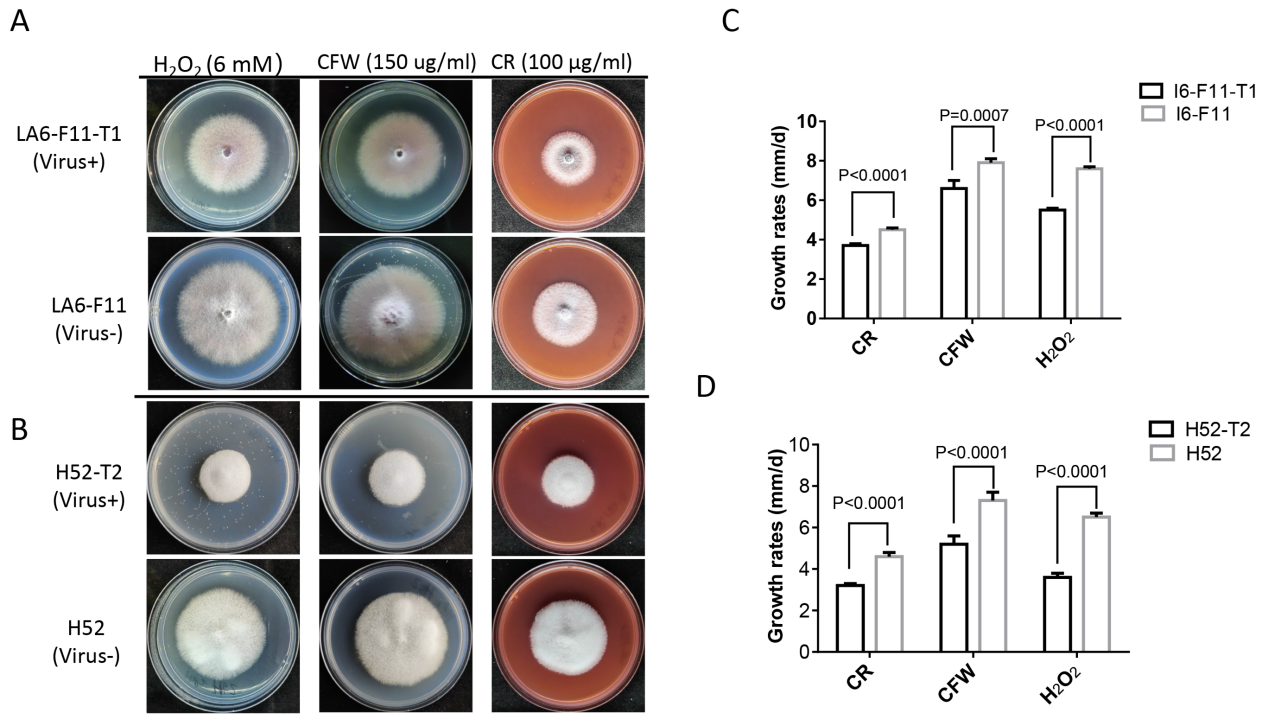


**Fig. S8 Effects of HadV1_LA6 on *Foc* morphology and growth.**

**(A and B)** Colony morphology of HadV1_LA6 re-introduced strains LA6-F11-T1 and H52-T2 compared to virus-free strains LA6-F11 and H52.

**(C and D)** Growth rates of the aforementioned strains; columns indicate the average growth rates of four independent cultures for each subisolate, with error bars represent standard deviation. Statistical analysis was conducted using an unpaired t-test.


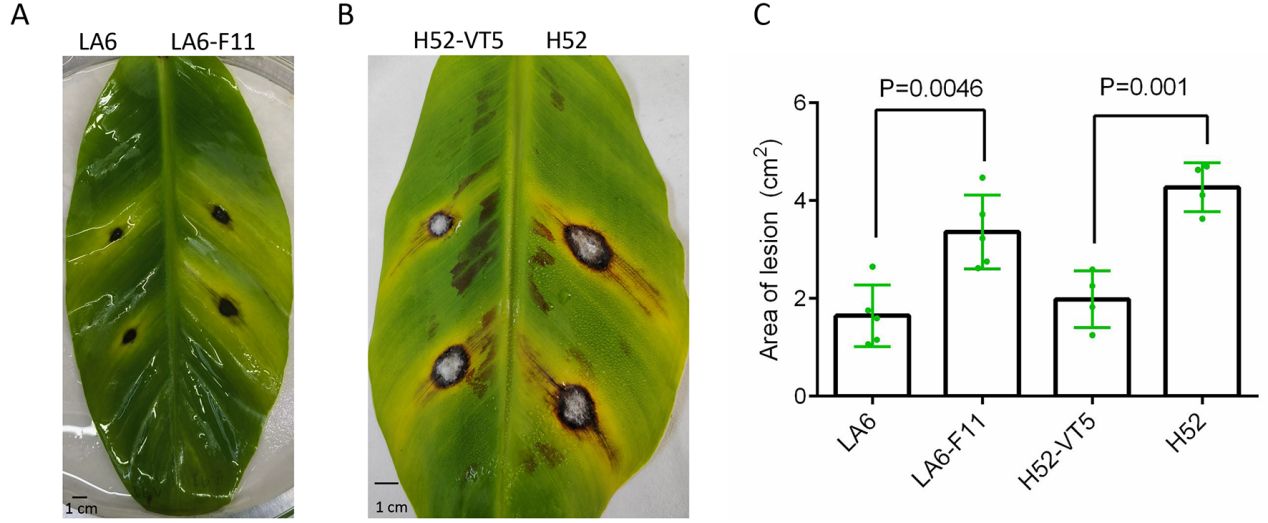


**Fig. S9 Virulence assay of the** **indicated strains on the detached banana seedling leaves (72 h post-inoculation at 28℃).**

**(A and B)** Representative symptoms on leaves following inoculation with the indicated strains. LA6: HadV1_LA6 infected strain, LA6-F11: virus-cured isolate, H52-VT5: horizontal transmission strain, H52: HadV1_LA6 free isolate H52.

**(C)** Lesion sizes induced by inoculation with the aforementioned strains; columns indicate the average size for each strain, error bars represent standard deviation and green dots indicate individual measurements. The differences between HadV1_LA6 infected and HadV1_LA6 free strains are statistically significant.


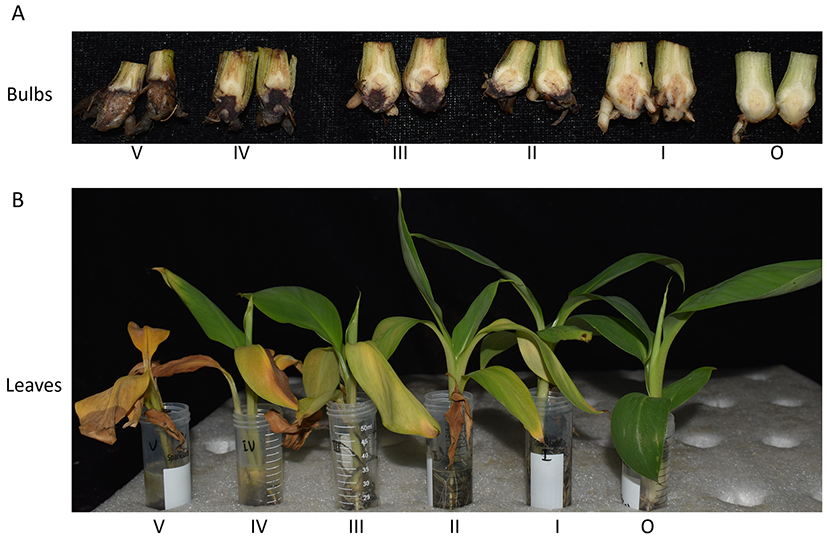


**Fig. S10 Value grade of *Fusarium* wilt of plant seedlings after inoculated *Foc* strains for 40 days in pot experiment.**

**(A)** Grading of rhizome browning from 0 (no symptoms) to IV (complete browning);

**(B)** Severity grading of leaf yellowing from 0 (no symptoms) to IV (complete yellowing).


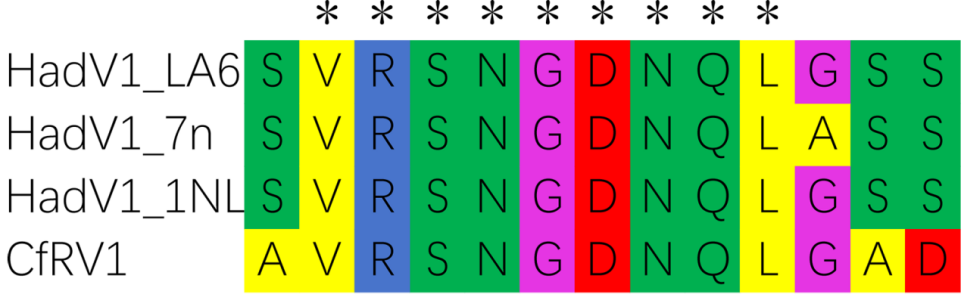


**Fig. S11** **Alignment of conserved RdRp motif C sequences of hadakavirids.** The aligned sequences were visualized using MEGA6.


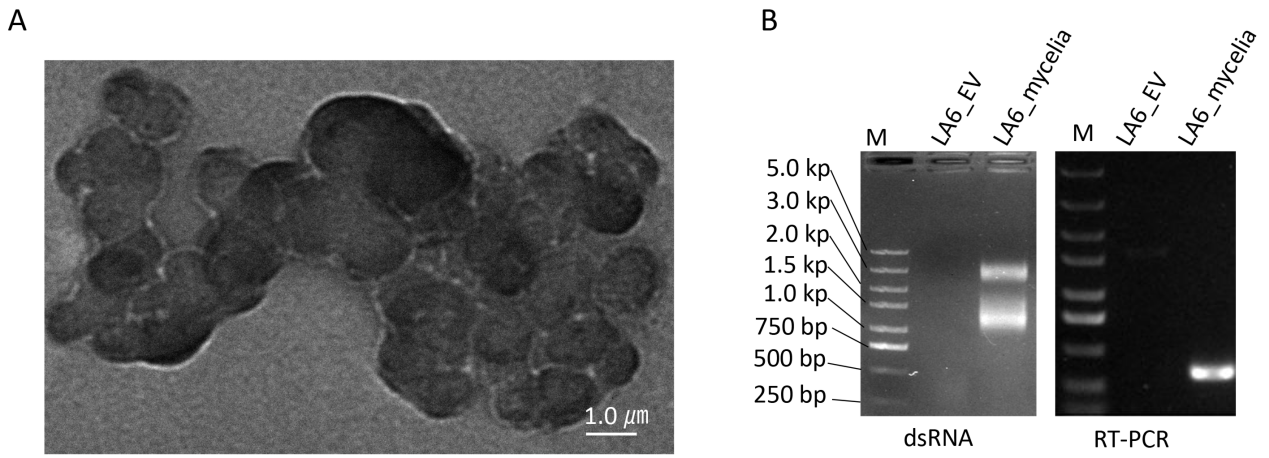


**Fig. S12 Detection of HadV1-LA6 in extracellular vesicles from *Foc* strain LA6.**

**(A)** Electron micrograph of extracellular vesicles.

**(B)** Detection of HadV1-LA6 using dsRNA extraction (left) and RT-PCR (right) from extracellular vesicles and mycelia, respectively. EV: extracellular vesicles. LA6 mycelia were used as the positive control.

**Reference**

1. Min-hui L, Xiong-tao Y, Hong-fei W, Jia-nuan Z, Ping-gen X, Zi-de J. 2012. Rapid Detection and Identification of *Fusarium oxysporum* f. sp. *cubense* Race 1 and Race 4. Scientia Agricultura Sinica 45:3971-3979. https://api.semanticscholar.org/CorpusID:86948414.
2. Dita MA, Waalwijk C, Buddenhagen I, Souza Jr M, Kema G. 2010. A molecular diagnostic for tropical race 4 of the banana fusarium wilt pathogen. Plant Pathol 59: 348-357. Doi: 10.1111/j.1365-3059.2009.02221.x.
3. Carvalhais LC, Henderson J, Rincon-Florez VA, O'Dwyer C, Czislowski E, Aitken EAB, Drenth A. 2019. Molecular Diagnostics of Banana Fusarium Wilt Targeting Secreted-in-Xylem Genes. Front Plant Sci 10: 547. doi: 10.3389/fpls.2019.00547.
